# Supplementary material for: Disinhibitory signaling enables flexible coding of top-down information in cortical networks
Source: PLoS Biol. 2026 Jul 2;24(7):e3003831. doi: 10.1371/journal.pbio.3003831 (PMC13327264; doi:10.1371/journal.pbio.3003831)
Supplement: Attachment — Submitted filename: 25_Aquino_Kim_Response_Letter_R1.pdf [file pbio.3003831.s025.pdf]

Reviewer #1: This paper uses extensive training and simulations with RNNs to investigate the interplay between task context and sensory coding. The authors describe how a disinhibitory motif arises as a result of training and study how this motif influences task performance and sensory representations. For that purpose, they use a few different variants of the RNN, as well as computational lesions. In addition, they use a publicly available experimental dataset, with which they carry out analyses that, as they report, substantiate their computational findings. Overall, the paper highlights the disinhibitory motif as a biologically plausible mechanism that is important for sensory processing, retaining of relevant information, and adaptive context integration.

I find the paper interesting, significant, and useful for the community. It is well written, and results are generally well substantiated by data and analyses. Below are several suggestions, which the authors might find useful for improving the paper.

We thank the reviewer for the thoughtful and positive evaluation of our work. We have carefully considered the reviewer's suggestions to further improve the clarity and impact of the manuscript (see below).

In addition, we apologize for the confusion caused by an inadvertent labeling error in the original manuscript. Specifically, the labels indicating pro vs. anti conditions and modality 1 vs. modality 2 were accidentally swapped in the original Fig. 4c–g and Fig. 6c–f. We have now corrected these labels and carefully revised the corresponding figure panels and captions. In addition, we have thoroughly reviewed and double-checked all related analyses and results to ensure their accuracy and internal consistency, and we have edited the relevant sections of the Results to explicitly reflect these corrections. Importantly, these labeling errors do not affect the underlying analyses, statistical outcomes, or conclusions of the study.

* Would it make sense to carry out perturbational analysis of E→E and E→I connections? The analysis of inhibitory connections in Figs. 4 and 6 is interesting and useful, but doing the same for excitatory connections should reveal more about their relative roles, or at the very least establish sort of a baseline against which perturbations of inhibitory connections can be considered.

We thank the reviewer for this insightful suggestion and fully agree that a systematic perturbational analysis of all four synaptic connection classes ($E \to E$, $E \to I$, $I \to E$, and $I \to I$) is important for clarifying their relative contributions. In response, we have now extended our lesion analyses to include outbound connections from excitatory neurons (i.e., $E \to E$ and $E \to I$), using the same selectivity-matched and crossed perturbation framework applied previously to inhibitory connections.

As shown in Response Fig. 1, for one-modality RNNs, perturbation of $E \to E$ connections produced minimal changes in performance across task conditions, indicating that recurrent excitation alone does not strongly determine task rule implementation. In contrast, lesioning $E \to I$ connections led to significant but comparatively modest performance impairments, and only when the lesions were selectivity-matched (i.e., $E_{\text{anti}} \to I_{\text{anti}}$). These findings are most likely due to weakened excitatory drive onto the rule-consistent inhibitory population, which reduces the efficacy of inhibitory competition and leads to partial performance degradation, particularly for the opposite rule.

For two-modality RNNs, we found that perturbations of both $E \to E$ and $E \to I$ connections did not produce significant changes in performance, regardless of whether lesions were selectivity-matched or crossed, or whether task or attention cues were fixed (Response Fig. 2–5). This suggests that in the more complex, multi-modal setting, excitatory outputs are even less critical for maintaining task-relevant context, consistent with a greater reliance on inhibitory circuit motifs to flexibly gate and multiplex information across modalities.

We have now included the below text in lines 321–330:

"To further assess the relative contributions of excitatory versus inhibitory outputs, we extended our lesion analyses to include selectivity-matched perturbations of excitatory-to-excitatory ($E \to E$) and excitatory-to-inhibitory ($E \to I$) connections. Across both one- and two-modality RNNs, perturbation of $E \to E$ connections produced minimal performance changes, while lesioning $E \to I$ connections led to modest impairments that were substantially smaller than those observed following disruption of $I \to I$ connectivity (Supplementary Fig. 8–12). These results indicate that, although excitatory projections contribute to shaping inhibitory competition, disinhibitory $I \to I$ circuitry remains the dominant mechanism supporting the maintenance and flexible deployment of task- and attention-related top-down information."

We have now included Response Fig. 1–5 as Supplementary Fig. 8–12.

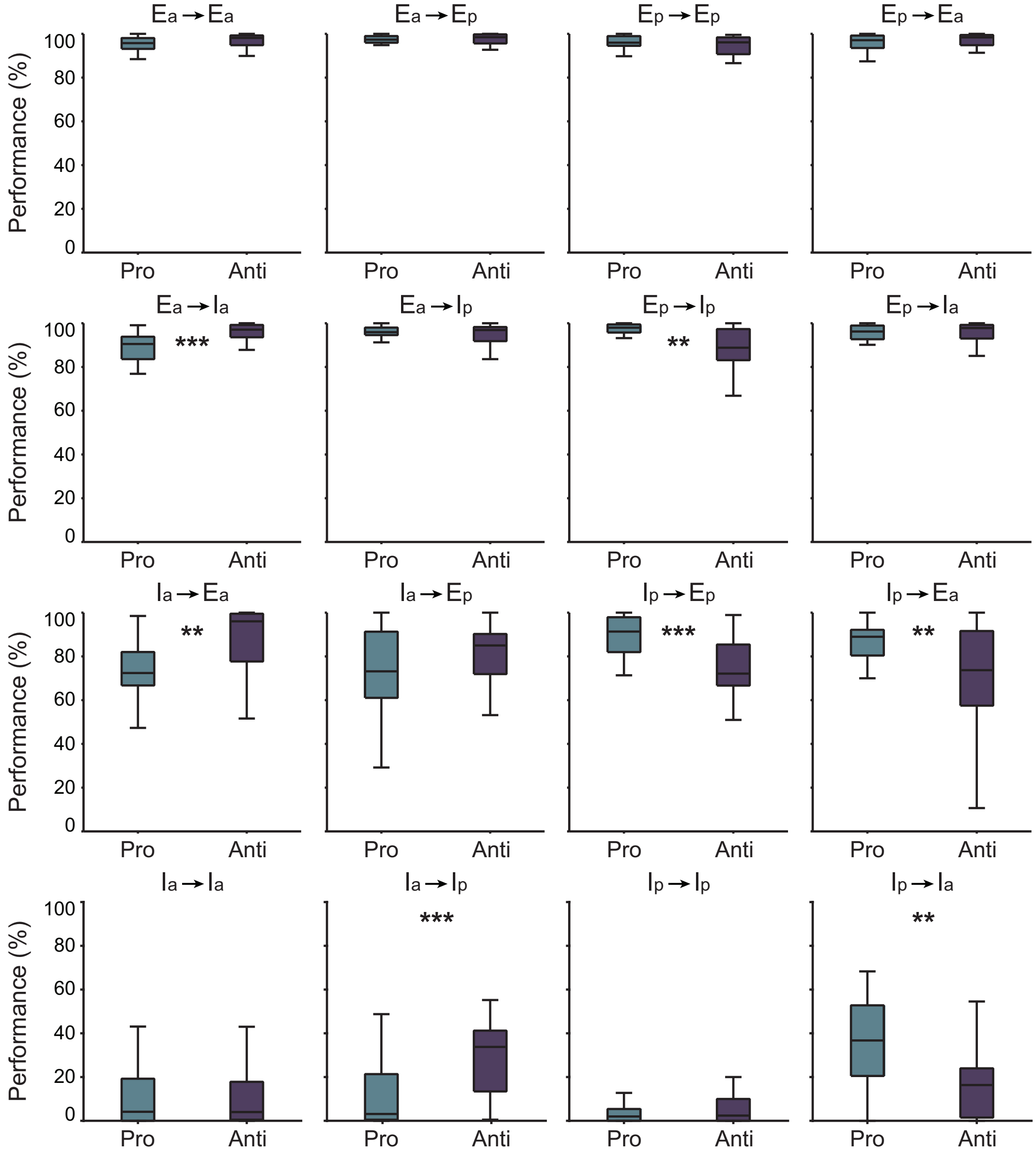

Response Fig. 1: **Perturbational analysis of excitatory and inhibitory synaptic connections in one-modality RNNs.** Task performance following selective lesioning of synaptic connections based on presynaptic/postsynaptic cell type and task selectivity. Rows correspond to different connection classes ($E \to E$, $E \to I$, $I \to E$, $I \to I$), and columns show lesions applied to selectivity-matched or crossed populations (anti vs. pro). Lesions were implemented by reducing the corresponding synaptic weights by 50%, and performance is shown separately for anti and pro trials. Boxplot: central lines, median; bottom and top edges, lower and upper quartiles; whiskers, 1.5 × interquartile range; outliers are not plotted. $^{**}P < 0.01$ and $^{***}P < 0.001$ by two-sided Wilcoxon rank-sum test.

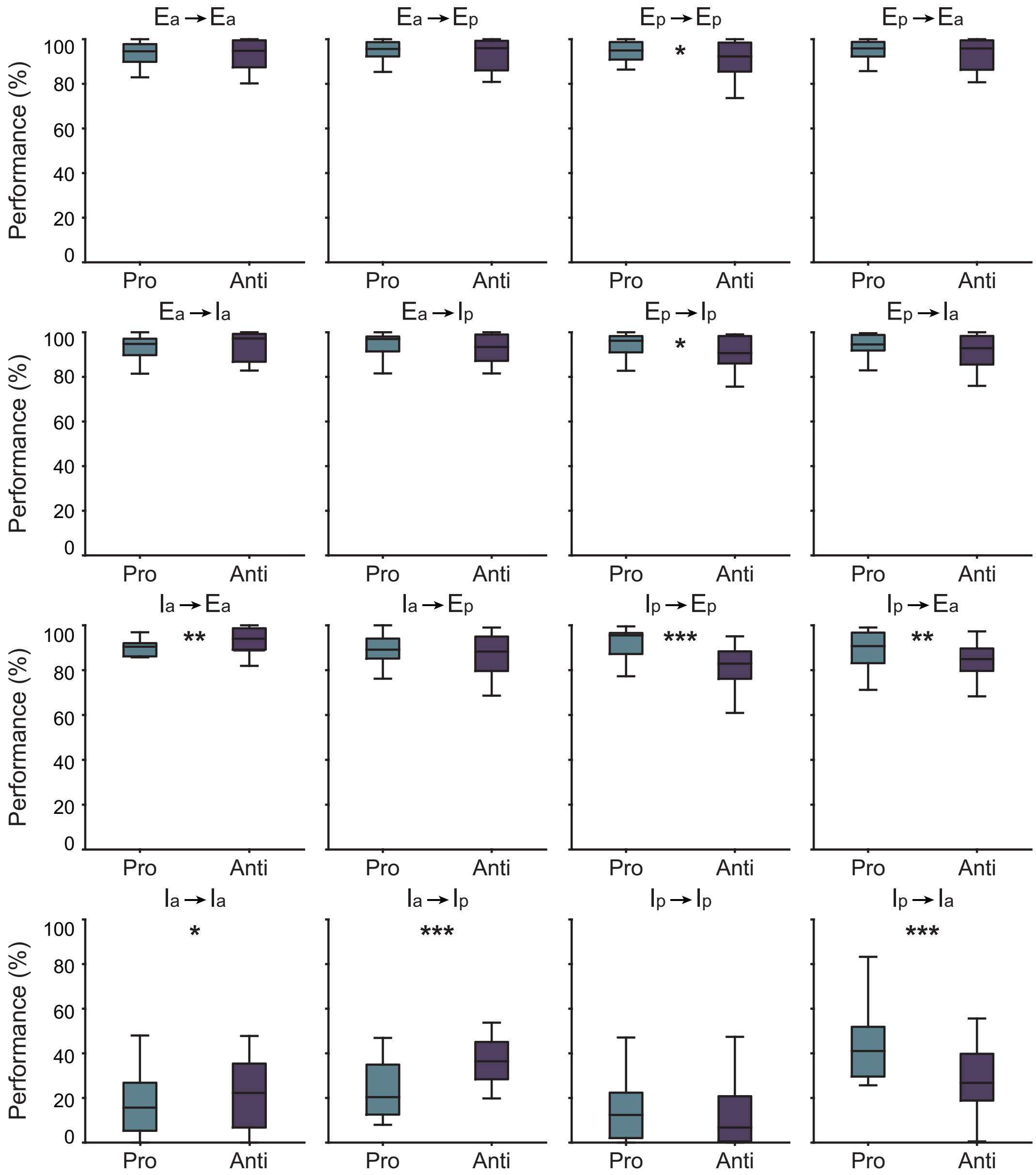

Response Fig. 2: **Perturbational analysis of excitatory and inhibitory synaptic connections in two-modality RNNs when modality 1 is attended.** Task performance following selective lesioning of synaptic connections in two-modality RNNs, with the attention cue fixed to modality 1. Figure organization follows the same layout and conventions as Supplementary Fig. 7. Boxplot: central lines, median; bottom and top edges, lower and upper quartiles; whiskers, 1.5 × interquartile range; outliers are not plotted. $*P < 0.05$, $**P < 0.01$, $***P < 0.001$ by two-sided Wilcoxon rank-sum test.

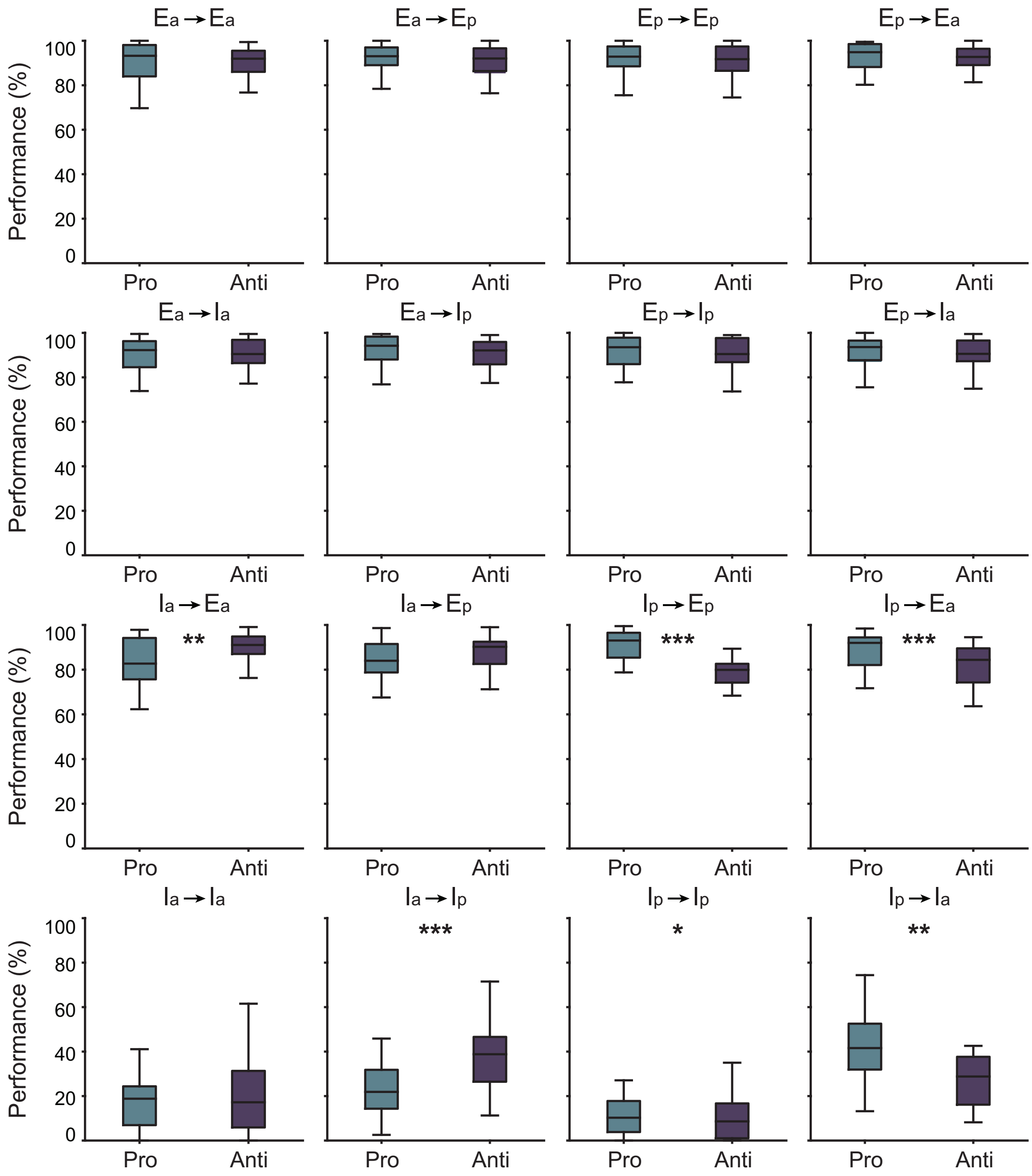

Response Fig. 3: **Perturbational analysis of excitatory and inhibitory synaptic connections in two-modality RNNs when modality 2 is attended.** Task performance following selective lesioning of synaptic connections in two-modality RNNs, with the attention cue fixed to modality 2. Figure organization follows the same layout and conventions as Supplementary Fig. 7. Boxplot: central lines, median; bottom and top edges, lower and upper quartiles; whiskers, 1.5 × interquartile range; outliers are not plotted. $*P < 0.05$, $**P < 0.01$, $***P < 0.001$ by two-sided Wilcoxon rank-sum test.

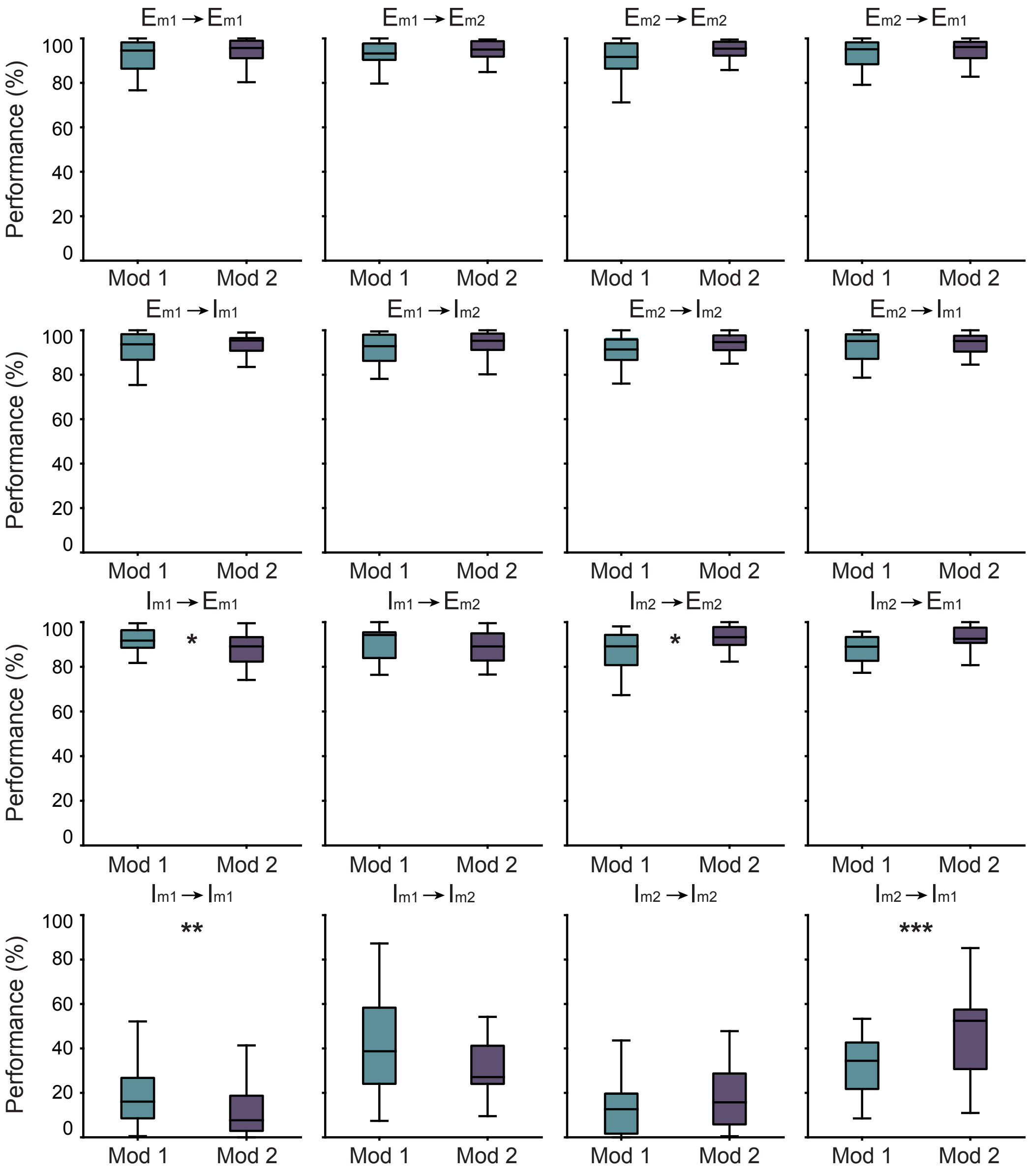

Response Fig. 4: **Perturbational analysis of excitatory and inhibitory synaptic connections in two-modality RNNs with the task signal fixed to the pro-DMS condition.** Task performance following selective lesioning of synaptic connections in two-modality RNNs, with the task cue fixed to the pro-DMS condition and performance compared between trials in which modality 1 or modality 2 was attended. Figure organization follows the same layout and conventions as Supplementary Fig. 7. Boxplot: central lines, median; bottom and top edges, lower and upper quartiles; whiskers, 1.5 × interquartile range; outliers are not plotted. $*P < 0.05$, $**P < 0.01$, $***P < 0.001$ by two-sided Wilcoxon rank-sum test.

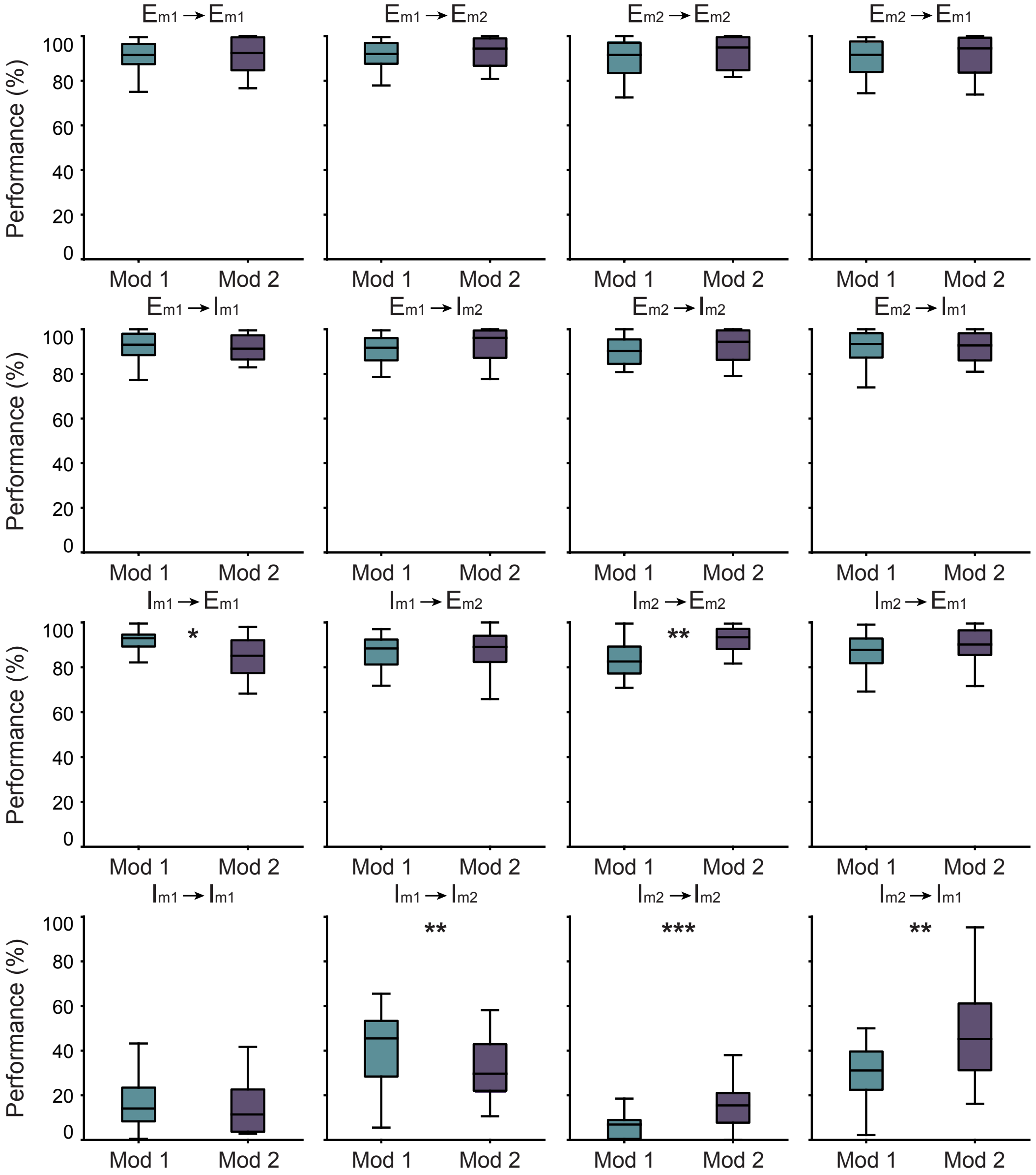

Response Fig. 5: **Perturbational analysis of excitatory and inhibitory synaptic connections in two-modality RNNs with the task signal fixed to the anti-DMS condition.** Task performance following selective lesioning of synaptic connections in two-modality RNNs, with the task cue fixed to the anti-DMS condition and performance compared between trials in which modality 1 or modality 2 was attended. Figure organization follows the same layout and conventions as Supplementary Fig. 7. Boxplot: central lines, median; bottom and top edges, lower and upper quartiles; whiskers, 1.5 × interquartile range; outliers are not plotted. $*P < 0.05$, $**P < 0.01$, $***P < 0.001$ by two-sided Wilcoxon rank-sum test.

* It would be useful to provide example plots of activity in the RNNs - perhaps heat maps, with time on the x-axis, neuron ID on the y-axis, and the level of activity of each neuron indicated by color. I am sure readers would like to see how those plots look like for the different tasks, conditions, and models presented. They could be added to each figure where a new task or model is introduced. Also, maybe in Fig. 1, it would be good to show an example of such activity plot early in the training and after training.

We thank the reviewer for this suggestion. We have now included example activity plots shown as neuron-by-time heatmaps, along with corresponding example RNN output signals, for representative trained RNNs as supplementary figures (Supplementary Fig. 1 and Supplementary Fig. 13). These figures are also shown below (Response Fig. 6 and Response Fig. 7). In response to Reviewer 2's comment regarding figure complexity and redundancy, we opted to include these examples as the supplementary figures.

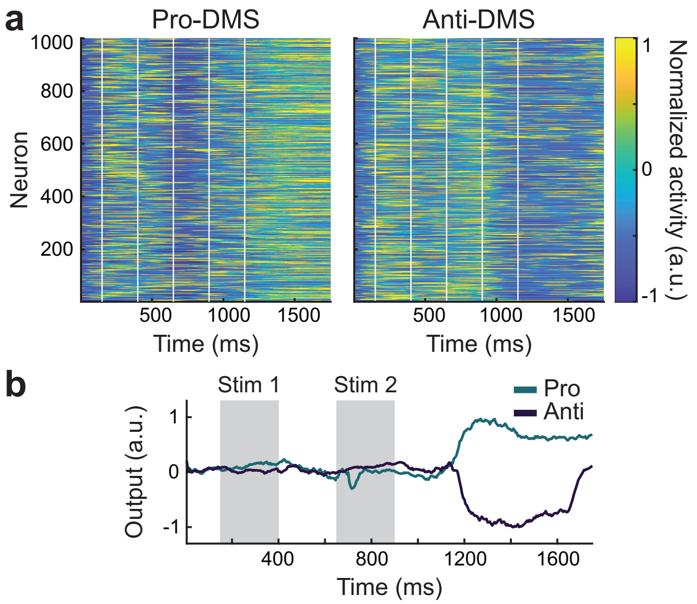

Response Fig. 6: **Example single-unit activity and output dynamics from a trained one-modality RNN. a.** Neuron-by-time heatmaps showing activity of all 1000 units from a representative single-module, one-modality RNN during Pro-DMS (left) and Anti-DMS (right) trials. For visualization purposes only, each neuron's activity was z-scored across time. White vertical lines demarcate key task epochs, including instruction, stimulus, delay, and response periods. **b.** Corresponding RNN output signals for Pro-DMS (teal) and Anti-DMS (purple) trials. Gray shaded regions indicate the presentation of stimulus 1 (Stim 1) and stimulus 2 (Stim 2). The output reflects the learned stimulus–response mapping conditioned on the task cue.

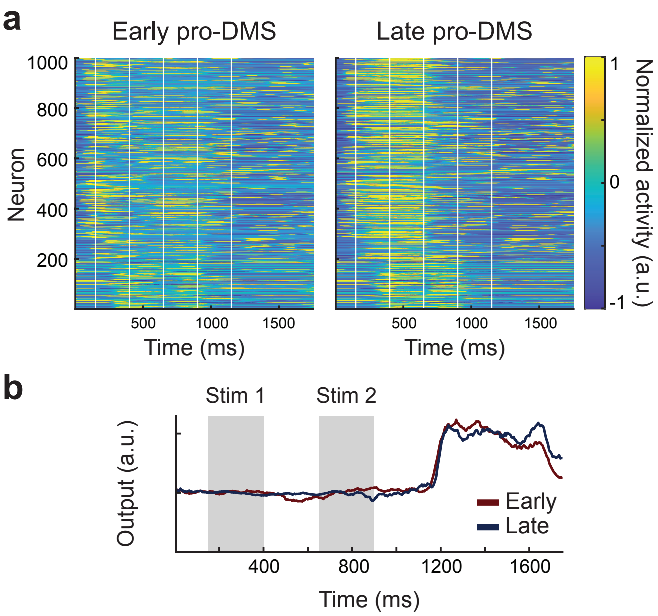

Response Fig. 7: **Example single-unit activity and output dynamics from a trained two-module, one-modality RNN. a.** Neuron-by-time heatmaps showing activity of all 1000 units from a representative two-module, one-modality RNN during Pro-DMS early (left) and late (right) instruction trials. For visualization purposes only, each neuron's activity was z-scored across time. White vertical lines demarcate key task epochs, including instruction, stimulus, delay, and response periods. **b.** Corresponding RNN output signals for Pro-DMS early (red) and late (blue) instruction trials. Gray shaded regions indicate the presentation of stimulus 1 (Stim 1) and stimulus 2 (Stim 2). The output reflects the learned stimulus–response mapping conditioned on the task cue.

* Lines 222-224: "Through dissection of the RNNs performing the one-modality and two-modality DMS tasks, we found that the optimized synaptic decay time constants for the excitatory units were significantly larger compared to the time constants of the inhibitory units". Also Fig. 3a,b and 5d,e.

I would recommend that the authors comment on how these observations compare with real biological data. One issue is that excitatory synaptic decay time constants tend to be shorter than inhibitory ones, e.g., in the mouse visual cortex, roughly by a factor of two. And the other is that typical values for these time constants are 10-30 ms (though both smaller and greater values are observed for some connection types), as opposed to 60-100 ms emerging in this work. See, for example, Campagnola et al., Science, 2022. While one should not demand too much biological realism from an RNN, it would be reasonable to explain the discrepancies and discuss why one should not worry about them too much.

We thank the reviewer for raising this important point. As the reviewer noted, prior experimental studies have shown that molecular synaptic decay time constants tend to be relatively fast for both excitatory and inhibitory receptors (e.g., AMPA, NMDA, GABA; see Campagnola et al., 2022; Zador & Dobrunz, 1997). In our RNNs, however, the optimized decay constants should be interpreted not as literal biophysical synaptic kinetics but rather as effective, functional time constants that emerge through training to support the task's computational demands.

Consistent with this interpretation, previous studies examining intrinsic neuronal time scales in cortical circuits and computational models have shown that excitatory and inhibitory populations

can exhibit long integration windows on the order of 100–200 ms, particularly in association cortex and recurrent models of cognitive processing (Murray et al., 2014; Wasmuht et al., 2018; Spaak et al., 2017; Wang, 2001). These longer intrinsic timescales arise from recurrent excitatory dynamics rather than fast synaptic receptor kinetics, and they allow excitatory neurons to maintain contextual information across delays. Thus, the longer effective time constants learned by the RNNs are consistent with the functional roles of excitatory and inhibitory populations observed in both empirical data and computational theory.

We have now included the below text (lines 248–250) to make this point clear:

"Importantly, these optimized decay time constants should be interpreted as effective functional timescales that emerge through training to support the task's computational demands."

* Line 445-446: "we observed a decrease in decoding accuracy following optogenetic silencing of V1 VIP neurons ($P < 0.05$, one-sided Kolmogorov-Smirnov test, Bonferroni corrected; Fig. 7c)".

Yes, but the difference is only 0.6 vs. 0.7 or so, and, more importantly, it is significant only during a very short time interval. To me, this seems like something one has to approach with some skepticism. I would recommend the authors to discuss the situation carefully and avoid making any substantial conclusions from this result.

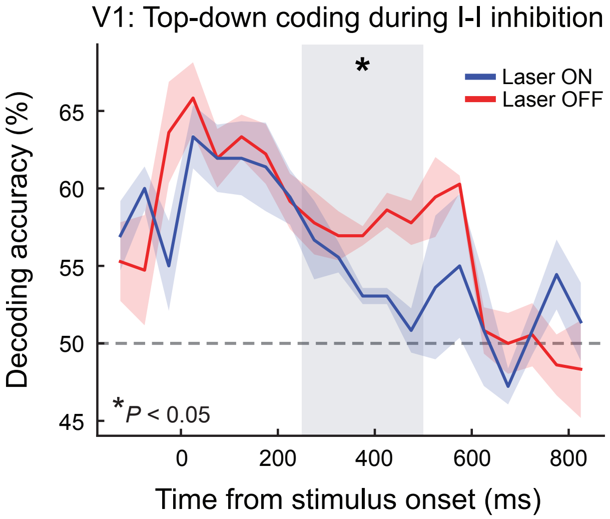

Response Fig. 8: **Updated disinhibitory decoding analysis**. SVM decoding of top-down signal during optogenetic I-I inhibition in V1, in laser on (orange) and laser off (blue) trials. Shaded area indicates laser on time window. P-value indicates significant difference in decoding across conditions ($P < 0.05$, one-sided Wilcoxon signed-rank test).

To address this point, we fully revisited the linear SVM decoding analysis of optogenetic silencing of VIP neurons within V1. To further align our analysis to Kirchberger et al. 2021, instead of selecting trials naively from the entire dataset, we restricted our analysis to the subset of orientation figure-ground effect trials. This subset was shown in the original manuscript (Kirchberger et al., 2021) to display the clearest figure-ground modulation in V1 neural populations.

Additionally, we performed a one-sided non-parametric test to determine whether the decoding performance was larger in the laser off condition, exclusively focused on the laser activation time window (from 0.25 s to 0.5 s following stim onset). Concretely, we are now analyzing a single 250 ms time window as a whole instead of testing several narrower time bins, which increased

the robustness of the analysis. We argue this created a clearer picture in favor of the effect of optogenetic I-I silencing in disrupting top-down coding in V1, exclusively while the laser is turned on. The new results are displayed in the new Fig. 7c, also included here (Response Fig. 8). We have also amended the methods and results sections to reflect these changes.

* Fig. 8 and associated text - this seems like an interesting result, but it is not clear how it connects with the rest of the paper, which is primarily about disinhibitory signaling in coding of top-down information. Perhaps there is an opportunity for the authors to investigate mechanisms of operation of their RNN in this case and compare that with what they did in the rest of the paper.

We agree with the reviewer that Fig. 8 would benefit from a clearer mechanistic connection to the core theme of the paper, which centers on the role of disinhibitory signaling in encoding and maintaining top-down information. To directly address this point, we performed additional perturbational (lesioning) analyses analogous to those used throughout the rest of the manuscript.

To address this, we performed additional perturbational analyses in which we systematically lesioned each of the four synaptic connection types ($E \to E$, $E \to I$, $I \to E$, and $I \to I$) in RNNs trained on the modified two-modality task with the retro-cue condition. Across all three attention-cue timing conditions (early, late, and retro), lesioning inhibitory-to-inhibitory connections by a 50% reduction led to the most pronounced decrease in the temporal discriminability of the first stimulus, whereas lesioning the other three synaptic classes produced substantially weaker effects (Response Fig. 9). These results demonstrate that, despite the altered task demands imposed by the retro-cue condition, the network continues to rely on disinhibitory $I \to I$ circuitry to maintain stimulus representations over time.

Accordingly, we have updated Fig. 8 to explicitly include the $I \to I$ lesioning results, and present the full lesioning analyses (Response Fig. 9) in a new supplementary figure (Supplementary Fig. 21). We have also revised the Results section to explicitly discuss these new analyses and clarify how the retro-cue findings in Fig. 8 relate to the broader disinhibitory mechanism identified throughout the study (lines 550–555):

"Carrying out systematic lesioning analyses revealed that reducing inhibitory-to-inhibitory ($I \to I$) connections led to a pronounced decrease in the temporal discriminability of the first stimulus (Fig. 8g–i), whereas lesioning the other synaptic classes produced substantially weaker effects (Supplementary Fig. 21). These results demonstrate that, despite the altered task demands imposed by the retro-cue condition, stimulus representations continue to rely critically on disinhibitory $I \to I$ circuitry for their maintenance."

We also added the following sentence in lines 558–560:

"Notably, this shift in strategy preserves the same underlying circuit mechanism, with disinhibitory inhibitory-to-inhibitory interactions remaining essential for stabilizing stimulus representations under dynamically changing attentional demands."

* I would suggest adding several references to recent work on the structure and function of cortical disinhibitory circuits, such as that with a large experimental dataset from the Allen Institute.

We thank the reviewer for the suggestions in strengthening our literature review. We have added several recent references to our introduction:

Garrett et al. Stimulus novelty uncovers coding diversity in survey of visual cortex. BioRxiv (2023),

Piet et al. Behavioral strategy shapes activation of the Vip-Sst disinhibitory circuit in visual cortex. Neuron (2024),

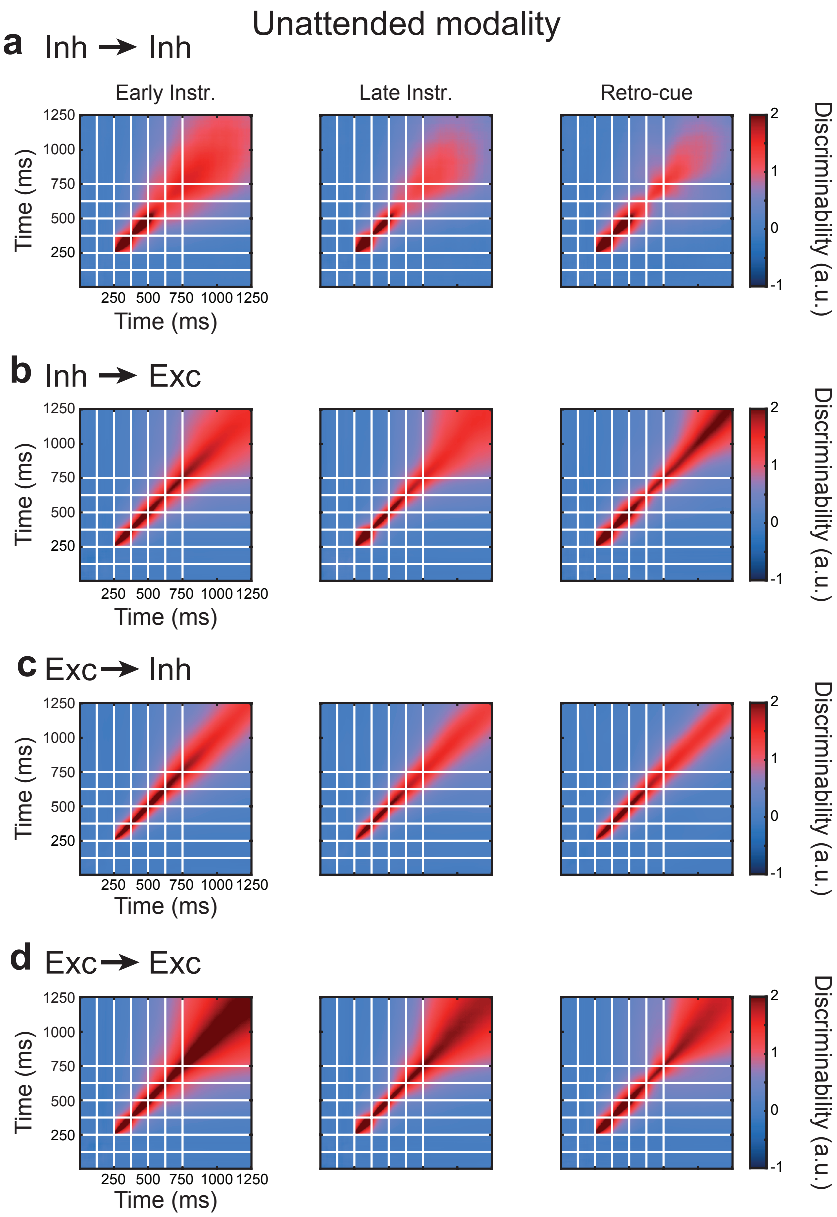

Response Fig. 9: **Effects of synaptic lesioning on unattended stimulus representations in two-module RNNs.** Average cross-temporal discriminability of the first stimulus identity of the unattended modality in two-module RNNs trained to perform the two-modality DMS task. Discriminability matrices are shown for trials in which the attention cue was presented early (left), late (middle), or as a retro-cue (right). Rows correspond to selective lesioning (50% reduction in synaptic weights) of different connection types: **a**. Inhibitory-to-inhibitory ($I \to I$). **b**. Inhibitory-to-excitatory ($I \to E$). **c**. Excitatory-to-inhibitory ($E \to I$). **d**. Excitatory-to-excitatory ($E \to E$) connections. White lines denote task epoch boundaries. Results are averaged across 20 trained RNNs.

Ito et al. Coordinated changes in a cortical circuit sculpt effects of novelty on neural dynamics. Cell Reports (2024),

Schulz et al. The generation of cortical novelty responses through inhibitory plasticity. eLife (2021),

Wu et al. Regulation of circuit organization and function through inhibitory synaptic plasticity. Trends in Neurosciences (2022).

Reviewer #2 (Katrin Franke signed her report): I believe this is a well-executed study that addresses an interesting question in sensory neuroscience. Specifically, understanding how context and top-down signals influence feedforward sensory processing is essential for explaining behavior, but this question is experimentally very challenging to approach. It typically requires selective silencing or manipulation of specific neural populations under well-controlled conditions, which can be difficult to achieve, especially at the level of precise microcircuits. Here, the authors take an elegant approach by using biologically constrained recurrent neural networks (RNNs) combined with circuit-level analyses of the model. This modeling framework allows for systematic and targeted investigation of circuit motifs—such as inhibitory-to-inhibitory connections—that may be difficult to test exhaustively in vivo. The inclusion of neural recordings from mouse visual cortex further strengthens the study by grounding the model findings in empirical data and demonstrating that similar circuit patterns may exist in the biological brain. While the results build on prior work and are generally consistent with current hypotheses about disinhibitory circuits and top-down modulation, the combination of computational and experimental approaches makes this study a valuable and well-integrated contribution to the field.

We thank the reviewer for the thoughtful and encouraging assessment of our study. We are especially grateful for the recognition of our combined computational and experimental approach, which we agree provides a unique opportunity to probe microcircuit motifs such as inhibitory-to-inhibitory connections.

In the interest of clarity and transparency, we would also like to note and correct an inadvertent labeling error in the original manuscript. Specifically, the labels indicating pro vs. anti conditions and modality 1 vs. modality 2 were accidentally swapped in the original Fig. 4c–g and Fig. 6c–f. We have now corrected these labels and carefully revised the corresponding figure panels and captions. In addition, we have thoroughly reviewed and double-checked all related analyses and results to ensure their accuracy and internal consistency, and we have edited the relevant sections of the Results to explicitly reflect these corrections. Importantly, these labeling errors do not affect the underlying analyses, statistical outcomes, or conclusions of the study. We apologize for any confusion this may have caused.

Major Points

Accessibility for Non-experts
The manuscript is well written and clearly motivated, but portions of the text and figures may be challenging for readers less familiar with computational modeling. For example, Figure 1 would benefit from a brief explanation in the main text about how the RNNs are trained and constrained (e.g., Dale's principle), and why this matters.

We thank the reviewer for this helpful suggestion. We agree that additional details on the RNN architecture and training constraints in the main text would improve accessibility for readers less familiar with computational modeling. To address this, we revised the relevant passage as follows:

From (lines 89–92):
"We utilized a gradient descent approach to train the RNN model, optimizing the recurrent connectivity weights, readout output weights, and synaptic decay time constants (see *Methods* for details). In addition, Dale's principle was enforced using the weight parametrization method introduced in [43]."

To:
"We trained the RNNs using a supervised gradient-descent procedure, in which the network learned to adjust its recurrent synaptic weights, output readout weights, and neuron-specific synaptic decay

time constants to perform the task accurately (Fig. 1; see *Methods*). To preserve biological interpretability, we enforced Dale's principle by constraining each unit to be either purely excitatory or purely inhibitory, using a sign-constrained weight parametrization [43]. These constraints ensure that learned network dynamics can be meaningfully related to cortical circuit motifs, enabling mechanistic interpretation of how specific inhibitory circuits support flexible encoding of top-down information."

In Figure 2a,b, interpreting the energy landscape plots was difficult, whereas the trajectory plots were more intuitive. It would help to clarify why both principal component space and latent space are used, how they differ, and how much variance each captures.

To clarify these points, we have included a more detailed explanation in the Results. The energy landscape plots and the trajectory plots are qualitative ways to visualize which portions of the neural network's activity landscape are more likely to be stable or have slower trajectories, following on Golub and Susillo, 2018, and Driscoll et al., 2024. The intuition behind the energy landscapes is that their lower points correspond to lower trajectory velocity. In these regions of the network's state space, we observe the formation of local energy minima, which can be slow points or fixed points. It is useful to visualize the transformation of these landscapes as a function of external inputs to the network, since that determines how specific instructions reshape the structure of the energy landscapes, and, therefore, the likelihood of each type of trajectory given a stimulus input. A quantitative understanding of these networks, analogous to explaining their variance, is obtained in the subsequent decoding analyses via decoding accuracy for the variables of interest.

Figure Complexity and Redundancy
Several figures (e.g., Figures 3 and 4) contain many similar panels and analyses. While I appreciate the authors' thoroughness, these sections were difficult to parse. Consider retaining only the most essential plots in the main figures, summarizing findings at a higher level, and moving more detailed analyses to the supplement.

We thank the reviewer for this helpful suggestion. In response, we have streamlined Fig. 3 and Fig. 4 by retaining only the panels that directly support the core conclusions, while moving more detailed and complementary analyses to supplementary figures. We believe these changes substantially improve clarity and readability while preserving the completeness of the analyses.

Clarification of Quantitative Measures
It wasn't clear to me what the decoding plots represent, is this mean performance across all model units? Would be great to plot a measure of variability, e.g. through 5 or 10 fold cross-validation. Is this how the decoders were evaluated? The concept of functional centrality, which is used to describe inhibitory units as network hubs, could also use a more intuitive explanation—what does this measure imply in terms of network function?

We provided clarification for the decoding and centrality analyses in the Methods section. The decoding plots represent single trial decoding accuracy for a signal of interest, which could be the task signal (pro vs. anti-DMS), the modality signal, or the stimulus identity. All model units of a given kind (inhibitory or excitatory) are included simultaneously in the SVM decoder as features, so this can be understood as population decoding instead of simple averaging across individual neurons. The measures of variability (standard error of the mean) are included as shaded areas around the decoding accuracy trajectories, which are currently computed in cross-validated fashion across 5 folds. We clarified these points in the methods and figure captions.

Regarding functional centrality, we used a metric of closeness within the network's graph to determine the degree to which each neuron is a hub of the network or not. Concretely, a node has high

centrality when it is, on average, only a few shortest steps away from all other nodes. Intuitively, a neuron is a good vantage point or information broadcast hub if communication follows shortest paths. We clarified this point in the methods.

Experimental Validation (Figure 7)
The use of mouse V1 data to test model predictions is valuable. However, the effect of VIP silencing in Figure 7c appears relatively modest, especially when contrasted with clearer differences in trajectory structure (Figures 7d-e). Some commentary on this discrepancy would be helpful, along with an indication of how much variance is explained by the latent space representations.

To address this point, we fully revisited the linear SVM decoding analysis of optogenetic silencing of VIP neurons within V1. To further align our analysis to Kirchberger et al. 2021, instead of selecting trials naively from the entire dataset, we restricted our analysis to the subset of orientation figure-ground effect trials. This subset was shown in the original manuscript (Kirchberger et al., 2021) to display the clearest figure-ground modulation in V1 neural populations.

Additionally, we performed a one-sided non-parametric test to determine whether the decoding performance was larger in the laser off condition, exclusively focused on the laser activation time window (from 0.25s to 0.5s following stim onset). Concretely, we are now analyzing a single 250ms time window as a whole instead of testing several narrower time bins, which increased the robustness of the analysis. We argue this created a clearer picture in favor of the effect of optogenetic I-I silencing in disrupting top-down coding in V1, exclusively while the laser is turned on. The new results are displayed in the new Figure 7c, also included in Response Fig. 8. We have also amended the methods and results sections to reflect these changes.

Task-Specificity and Generalization
The retro-cue condition is a nice addition that shows flexibility in the model's behavior. However, it raises the question of how specific the findings are to this particular task design. I would encourage the authors to discuss whether the emergence of disinhibitory motifs might generalize to other forms of top-down modulation (e.g., reward-driven, predictive, or more naturalistic settings).

We thank the reviewer for this thoughtful comment. We agree that the introduction of the retro-cue condition naturally raises the question of whether the observed circuit mechanisms are specific to this task design or reflect a more general computational principle.

To address this, we performed additional lesion analyses in the retro-cue condition, which revealed that despite the altered task demands and increased requirement to maintain both attended and unattended stimulus representations over time, network performance continued to depend critically on disinhibitory inhibitory-to-inhibitory ($I \to I$) circuitry. In particular, lesioning $I \to I$ connections again produced the largest and most consistent impairments in performance and representational stability, indicating that the reliance on disinhibitory motifs is preserved even under substantially modified temporal and attentional demands (Response Fig. 9).

Accordingly, we have updated Fig. 8 to explicitly include the $I \to I$ lesioning results, and present the full lesioning analyses (Response Fig. 9) in a new supplementary figure (Supplementary Fig. 21). We have also revised the Results section to explicitly discuss these new analyses and clarify how the retro-cue findings in Fig. 8 relate to the broader disinhibitory mechanism identified throughout the study (lines 550–555):

"Carrying out systematic lesioning analyses revealed that reducing inhibitory-to-inhibitory ($I \to I$) connections led to a pronounced decrease in the temporal discriminability of the first stimulus (Fig. 8g–i), whereas lesioning the other synaptic classes produced substantially weaker effects (Supplementary Fig. 21). These results demonstrate that, despite the altered task demands imposed by

the retro-cue condition, stimulus representations continue to rely critically on disinhibitory $I \to I$ circuitry for their maintenance."

We have also included the following additional paragraph in the Discussion section (lines 597–609) to discuss additional experimental studies that highlight the importance of disinhibitory circuits in other forms of top-down modulation:

"Although the retro-cue condition introduces task demands that differ from the standard delayed match-to-sample structure, our additional lesion analyses demonstrate that the same disinhibitory inhibitory-to-inhibitory circuitry remains critical for maintaining stimulus representations under these altered temporal and attentional constraints. This suggests that the emergence of disinhibitory motifs in our models is not specific to a particular task configuration, but instead reflects a more general circuit mechanism for stabilizing and selectively prioritizing internal representations in the presence of dynamically changing top-down demands. In line with this, prior experimental and computational studies have shown that reward expectation and value signals strongly modulate cortical processing through inhibitory interneuron populations, often via disinhibitory pathways that gate sensory or mnemonic representations in a context-dependent manner [90–93]. Within this broader framework, our results suggest that disinhibitory $I \to I$ motifs may constitute a canonical circuit mechanism through which diverse top-down signals can dynamically regulate information flow and representational stability across cortical hierarchies."

Biological Interpretation
The link between the modeled inhibitory units and known interneuron types (e.g., VIP→SST motifs) could be expanded. This would help readers better connect the RNN findings to cortical microcircuitry. A more detailed discussion of biological implementation would be appreciated—especially for readers coming from an experimental background.

We expanded on the connection between artificial inhibitory units and biological interneurons, along with disinhibitory motifs, in the discussion.

Consistent with our findings on the central role of inhibitory-inhibitory connections, recent animal studies have identified disinhibitory circuit motifs facilitating flexible routing of sensory information. Specifically, the canonical VIP-SST disinhibitory circuit in cortex is a candidate for the biological implementation of the top-down regulatory mechanism elicited by inhibitory-inhibitory connections that arose in our artificial models. While VIP neurons are known to receive long-range feedback from frontal areas, and to be flexibly modulated by task demands, one of their roles in cortical circuitry is to inhibit SST expressing neurons, which are inhibitory neurons themselves. Since SST neurons target excitatory pyramidal neurons in visual circuitry, the end goal of this circuitry may be to allow for a regulated integration of top-down feedback into disinhibited excitatory sensory neurons.

Reviewer #3: In this manuscript, the authors studied recurrent neural networks (RNNs) trained to perform context-dependent delay match-to-sample tasks, where the correct response depends on the combination of sensory stimulus, task cue (pro or anti), and in some cases, the attentional cue (attend to modality 1 or 2). The RNNs have separate excitatory and inhibitory populations, and the authors also developed two-module RNNs where each module receives input about sensory stimulus and contextual cue, respectively.

The authors mainly focused on the function of the inhibitory populations in the RNNs, in terms of 1) how their activity differentially represent the task context when compared with that of the excitatory population, and 2) the impact on task performance when inhibitory synapses are "lesioned" (set to lower strength) in the network. The authors concluded that inhibitory neurons, as well as inhibitory-to-inhibitory connections, play a more important role in representing contextual signals, which is consistent with recent experimental findings that VIP inhibitory neurons, which target other inhibitory neurons, mediate contextual information. While this message is interesting and reported by an increasing number of experiments, I feel the analyses performed are not systematic and thorough enough to support this conclusion. For example, the authors showed that lesioning inhibitory-to-inhibitory (I to I) connections leads to an impairment in contextual behavioral performance, but did not do enough control analyses to show that lesioning other connections (E to E, E to I, I to E) leads to *less* performance deficit.

In addition, I would hope that a modeling study can reveal something beyond agreement with experimental data. The manuscript did not really touch on the deeper mechanistic questions, such as "why inhibitory neurons (and I to I connections) are more important in contextual tasks", or "what are the functional benefits of it". Below are more detailed comments:

We thank the reviewer for their careful reading of the manuscript and for the thoughtful, detailed critique. We appreciate the reviewer's emphasis on the need for more systematic control analyses and deeper mechanistic insight, which helped us substantially strengthen the rigor and interpretability of our results.

In the interest of clarity and transparency, and in direct relation to the reviewer's comment (c.), we would like to note and correct an inadvertent labeling error in the original manuscript. Specifically, the labels indicating pro vs. anti conditions and modality 1 vs. modality 2 were accidentally swapped in the original Fig. 4c–g and Fig. 6c–f. We have now corrected these labels and revised the corresponding figure panels and captions accordingly. In addition, we have carefully reviewed and double-checked all related analyses and results to ensure their accuracy and internal consistency, and we have edited the relevant sections of the Results to explicitly reflect these corrections. Importantly, these labeling errors do not affect the underlying analyses, statistical outcomes, or conclusions of the study, and we apologize for any confusion this may have caused.

1) The authors showed that the task and attention modality could be decoded better in the inhibitory neurons compared to excitatory neurons (Fig 3c-g; Fig 5h, i). This is interesting but could the authors explore a bit more what is driving this difference. Is it simply because I cells have a higher firing rate, and therefore a higher signal-to-noise ratio?

We thank the reviewer for this thoughtful question. We think inhibitory neurons act as context-setting control nodes whose activity summarizes the current task state by coordinating competition and suppression across excitatory subpopulations. This interpretation is consistent with our centrality analyses (Fig. 3e–h), which show that inhibitory neurons become functional hubs following top-down cue presentation, as well as with the lesion results demonstrating that disrupting inhibitory outputs, especially $I \to I$ connectivity, has disproportionate effects on contextual representations and performance.

2) Some comments related to the model lesion results in Figures 4 and 6.
a. The authors wanted to conclude that I to I connections are most important in task performance by showing that lesioning I to I connections lead to a significant decrease in performance. However, no comparison was made e effect of lesioning E to I and E to E connections, as well as I to E connections in some cases. Without these control analyses, it is difficult to see that I to I connections play an *especially* important role.

We thank the reviewer for this insightful suggestion and fully agree that a systematic perturbational analysis of all four synaptic connection classes ($E \to E$, $E \to I$, $I \to E$, and $I \to I$) is important for clarifying their relative contributions. In response, we have now extended our lesion analyses to include outbound connections from excitatory neurons (i.e., $E \to E$ and $E \to I$), using the same selectivity-matched and crossed perturbation framework applied previously to inhibitory connections.

As shown in Response Fig. 1, for one-modality RNNs, perturbation of $E \to E$ connections produced minimal changes in performance across task conditions, indicating that recurrent excitation alone does not strongly determine task rule implementation. In contrast, lesioning $E \to I$ connections led to significant but comparatively modest performance impairments, and only when the lesions were selectivity-matched (i.e., $E_{\text{anti}} \to I_{\text{anti}}$). These findings are most likely due to weakened excitatory drive onto the rule-consistent inhibitory population, which reduces the efficacy of inhibitory competition and leads to partial performance degradation, particularly for the opposite rule.

For two-modality RNNs, we found that perturbations of both $E \to E$ and $E \to I$ connections did not produce significant changes in performance, regardless of whether lesions were selectivity-matched or crossed, or whether task or attention cues were fixed (Response Fig. 2–5). This suggests that in the more complex, multi-modal setting, excitatory outputs are even less critical for maintaining task-relevant context, consistent with a greater reliance on inhibitory circuit motifs to flexibly gate and multiplex information across modalities.

We have now included the below text in lines 321–330:

"To further assess the relative contributions of excitatory versus inhibitory outputs, we extended our lesion analyses to include selectivity-matched perturbations of excitatory-to-excitatory ($E \to E$) and excitatory-to-inhibitory ($E \to I$) connections. Across both one- and two-modality RNNs, perturbation of $E \to E$ connections produced minimal performance changes, while lesioning $E \to I$ connections led to modest impairments that were substantially smaller than those observed following disruption of $I \to I$ connectivity (Supplementary Fig. 8–12). These results indicate that, although excitatory projections contribute to shaping inhibitory competition, disinhibitory $I \to I$ circuitry remains the dominant mechanism supporting the maintenance and flexible deployment of task- and attention-related top-down information."

We have now included Response Fig. 1–5 as Supplementary Fig. 8–12.

b. The authors performed the lesion by decreasing the connection between *selective* neurons. To ensure a fair comparison, it would be better to look at the fraction of selective E and I neurons, to exclude the possibility that lesioning I to I connections has a larger effect simply because a larger fraction of connections was lesioned.

We thank the reviewer for this important point. To address the concern that the larger behavioral impact of lesioning $I \to I$ connections might simply reflect a larger fraction of selective connections being perturbed, we explicitly quantified the number of outgoing synaptic connections across cell types. As shown in Response Fig. 10, for the one-modality single-module RNNs, inhibitory neurons in fact have fewer outgoing connections overall compared to excitatory neurons, and critically, $I \to I$

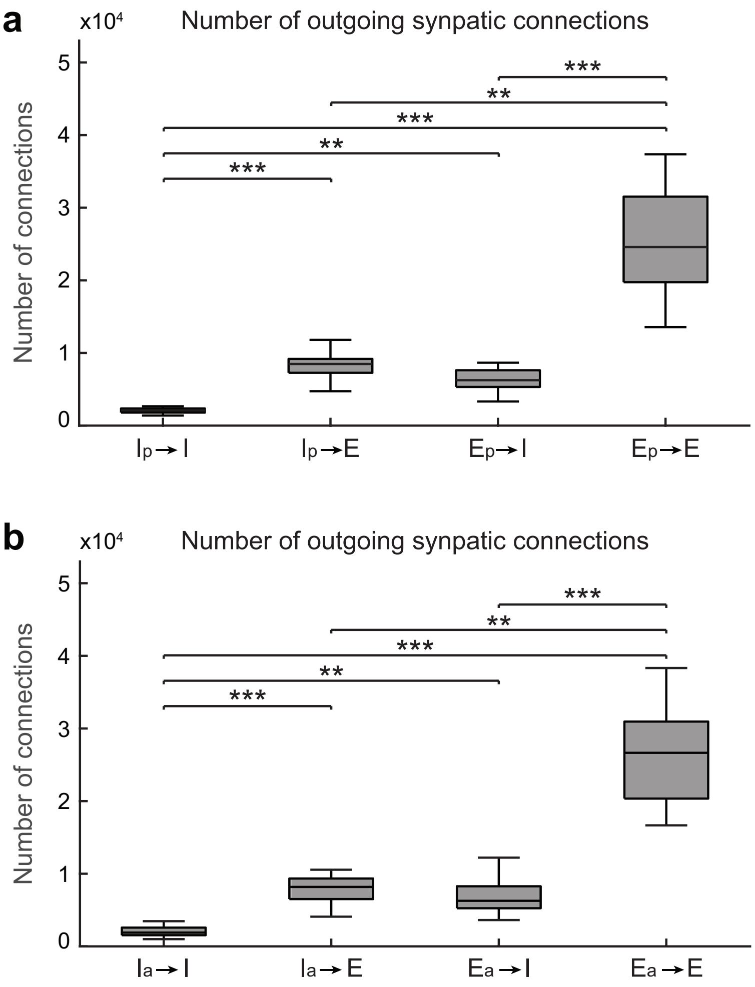

Response Fig. 10: **Quantification of outgoing synaptic connections in single-module one-modality RNNs.** Boxplots show the total number of outgoing synaptic connections for task-selective excitatory and inhibitory neuron populations across 20 single-module RNNs trained to perform the one-modality DMS task. Top: outgoing connections from pro-DMS-selective populations; bottom: outgoing connections from anti-DMS-selective populations. For each condition, outgoing connections are grouped by presynaptic and postsynaptic cell type ($I \to I$, $I \to E$, $E \to I$, $E \to E$). Boxplot: central lines, median; bottom and top edges, lower and upper quartiles; whiskers, 1.5 × interquartile range; outliers are not plotted. Statistical comparisons were performed using a Kruskal–Wallis test followed by Dunn's post hoc multiple-comparisons test with Dunn's multiple comparison test. Significance levels are indicated as * $P < 0.05$, ** $P < 0.01$, and *** $P < 0.001$.

connections constitute the smallest fraction of outgoing synapses relative to $I \to E$, $E \to I$, and $E \to E$ connections. Thus, lesioning $I \to I$ connections does not remove a disproportionate number of synapses. Despite this, selectively weakening $I \to I$ connections produces the most pronounced impairments in task performance. Importantly, the same trend holds for the two-modality RNNs when we quantify modality-specific selective neurons, indicating that the strong behavioral effects of $I \to I$ lesions cannot be explained by differences in the fraction of selective connections lesioned, but instead reflect a disproportionately important functional role of disinhibitory inhibitory-to-

inhibitory circuitry in encoding and maintaining top-down information.

We now include Response Fig. 10 as a supplementary figure and make this point in the main text (lines 315–318):

"Importantly, the disproportionately large behavioral effects observed following lesioning of task cue-specific $I \rightarrow I$ connections cannot be explained by a greater number of connections being perturbed, as $I \rightarrow I$ synapses constitute the smallest fraction of outgoing connections compared to other synaptic classes (Supplementary Fig. 6)."

c. In Figures 4 and 6, lesioning $Ia \rightarrow Ip$ connections impair anti-DMS trials more, and vice versa. Could the authors explore further why this is the case? Is it because Ip neurons inhibit Ea neurons? On the other hand, the opposite trend is true when it comes to modality 1 and 2 (Fig 4f, g). For example, lesioning the $I_{m1} \rightarrow I_{m2}$ connections has a larger impact on performance for modality 1 trials). Why is this the case? Since the authors already have classified different neuron types, maybe it is worthwhile to look at the structure in the wiring between different neuron populations.

We thank the reviewer for raising this point and for the opportunity to clarify the apparent asymmetries in the lesion effects shown in Fig. 4 and Fig. 6. This issue was directly related to an inadvertent swapping of the pro vs. anti and modality 1 vs. modality 2 labels in the original figures (now corrected). With the corrected labeling, the observed lesion effects follow a consistent and interpretable pattern. Specifically, lesioning $I_a \rightarrow I_p$ connections preferentially impairs pro-DMS performance, whereas lesioning $I_p \rightarrow I_a$ connections preferentially impairs anti-DMS performance. This pattern is consistent with the interpretation that disinhibitory signaling onto the rule-relevant inhibitory population is selectively disrupted—for example, $I_a \rightarrow I_p$ lesions weaken inhibitory support for pro-DMS signaling, leading to degraded pro-DMS performance.

An analogous relationship holds for the modality-specific lesions. With the corrected labels, lesioning $I_{m1} \rightarrow I_{m2}$ connections preferentially impairs modality 2 trials, while lesioning $I_{m2} \rightarrow I_{m1}$ connections preferentially impairs modality 1 performance. Thus, across both task-rule and modality dimensions, crossed inhibitory lesions consistently disrupt performance for the condition whose disinhibitory support is compromised. Together, these results indicate that the apparent inconsistencies noted by the reviewer were driven by the labeling error, and that once corrected, the lesion effects align with a coherent disinhibitory gating mechanism in which inhibitory-to-inhibitory connectivity selectively stabilizes the currently relevant rule or modality. We have now revised the figures (Fig. 4 and Fig. 6) and corresponding Results sections. We again thank the reviewer for this comment and apologize for any confusion this may have caused.

d. The authors performed the lesion by reducing the connection strength by 50%. Could the authors motivate why they chose 50% in particular? Is there some experimental evidence for choosing this number, or are the results robust across different levels of reduction?

We thank the reviewer for this important question. The choice to reduce synaptic connection strength by 50% was intended as a moderate perturbation that is strong enough to reveal functional dependence on specific circuit motifs, while avoiding complete ablation that could trivially disrupt network dynamics or lead to ceiling effects.

3) If the authors indeed found that excitatory connections are less important (which I think still needs more systematic evaluation, as mentioned in the previous point), what are the functional benefits of inhibitory neurons and I to I connections being more important in contextual tasks?

We thank the reviewer for this important conceptual question. As shown in Response Fig. 1–5, our results suggest that inhibitory neurons, in particular inhibitory-to-inhibitory ($I \rightarrow I$) connectivity,

provide distinct functional benefits for contextual and flexible tasks. Based on these findings, together with prior experimental results from Kirchberger et al., we hypothesize that a key functional advantage of inhibitory circuitry is its ability to selectively gate and stabilize internal representations without directly driving network output. Disinhibitory $I \rightarrow I$ motifs enable the network to rapidly reconfigure which excitatory subpopulations are active or suppressed based on contextual signals, thereby flexibly prioritizing task-relevant information while minimizing interference from competing representations. This form of control is especially well suited for contextual tasks, where the same sensory input must be interpreted differently depending on task rules, attention, or timing.

We have now expanded the Discussion to include additional studies highlighting the role of inhibitory signaling in top-down modulation (lines 597–609):

"Although the retro-cue condition introduces task demands that differ from the standard delayed match-to-sample structure, our additional lesion analyses demonstrate that the same disinhibitory inhibitory-to-inhibitory circuitry remains critical for maintaining stimulus representations under these altered temporal and attentional constraints. This suggests that the emergence of disinhibitory motifs in our models is not specific to a particular task configuration, but instead reflects a more general circuit mechanism for stabilizing and selectively prioritizing internal representations in the presence of dynamically changing top-down demands. In line with this, prior experimental and computational studies have shown that reward expectation and value signals strongly modulate cortical processing through inhibitory interneuron populations, often via disinhibitory pathways that gate sensory or mnemonic representations in a context-dependent manner [90–93]. Within this broader framework, our results suggest that disinhibitory $I \rightarrow I$ motifs may constitute a canonical circuit mechanism through which diverse top-down signals can dynamically regulate information flow and representational stability across cortical hierarchies."

4) The authors mentioned that in the one module network, the synaptic decay time constants for the excitatory units were significantly larger compared to those of the inhibitory units. The same is true for the non-sensory module of the two-module network, but the opposite was found for the sensory module. These results seem interesting and may warrants further exploration/discussion. For example, are they related to the functional difference between the E and I neurons in the model? Are there some functional benefits for this difference in time constants?

While these questions still warrant further directed study, we can argue in favor of possible interpretations for the role of heterogeneous time scales across excitatory and inhibitory neurons. Longer excitatory time constants in the sensory module may support integration over longer times, beneficial to context integration via top-down feedback to sensory areas. Faster excitation relative to inhibition, on the other hand, could support rapid sensory stimulus encoding. In the non-sensory module, more closely related to the final decision process, it is possible that an inverted encoding-decoding dynamic is at play. Decision making often requires integrating evidence over time (e.g. through drift-diffusion mechanisms), which could functionally necessitate longer effective integration windows. Slower excitatory synaptic dynamics are often incorporated to ensure that network dynamics smooth out noisy inputs and accumulate information gradually.

We added a couple of sentences to the Results section to reflect these arguments.

5) In the two-module network, the authors found that that the inhibitory connections are stronger in the non-sensory module compared to the sensory module, which is consistent with experimental data showing an increase in the strength of inhibition for higher-order cortices. It is nice that the model fits the data, but it would be nicer if the authors could offer some insights on any potential functional benefits this different in inhibition strength brings.

Leveraging the recent literature on excitation-inhibition balance, we can argue in favor of a possible interpretation for these findings. Stronger inhibition in the decision areas allows tighter control of excitation, which supports precise modulation of internal computations that depend on context and goals rather than direct sensory drive. In higher-order modules where computations mainly rely on recurrent internal dynamics (e.g. evidence accumulation, rule maintenance), stronger inhibition improves stability and prevent excessive activity. The recent literature suggests such a stabilizing role for inhibition in the context of strongly connected excitatory populations (Roach, Churchland, and Engel, 2023), expanding the parameter space where decision-making circuits can operate without instability.

We added a couple of sentences to the Results section to reflect these arguments.

Minor comments:
1) The authors wrote in the introduction that "... However, these studies [of RNN modeling] primarily examine scenarios in with task goals and contextual cues remain fixed over time." This is not accurate. There are many studies that train RNNs to perform contextual tasks. Examples include

* Mante, V., Sussillo, D., Shenoy, K. V., & Newsome, W. T. (2013). Context-dependent computation by recurrent dynamics in prefrontal cortex. nature, 503(7474), 78-84.

* Yang, G. R., Joglekar, M. R., Song, H. F., Newsome, W. T., & Wang, X. J. (2019). Task representations in neural networks trained to perform many cognitive tasks. Nature neuroscience, 22(2), 297-306.

* Liu, Y., & Wang, X. J. (2024). Flexible gating between subspaces in a neural network model of internally guided task switching. Nature communications, 15(1), 6497.

We have revised our introduction to accurately represent the literature and included these references.

2) Also in the introduction, the authors mentioned: "This results in temporally rich and highly dynamic decision-making, a phenomenon that has proven challenging to investigate in artificial neural network models, including RNNs". Could the authors provide some citations that support this claim?

We changed our affirmation to highlight the usefulness of RNNs in studying dynamic systems, including a comprehensive review on this topic:

Still, further work is required to understand how top-down information and bottom-up stimuli are integrated to efficiently represent current task demands. Importantly, these top-down factors, including selective attention, task contexts, and prior knowledge, can exhibit distinct temporal structures, imposing varied but complementary guiding rules on how sensory input should be processed. This results in temporally rich and highly dynamic decision-making, a phenomenon that can be modeled with artificial neural networks, including RNNs (Vyas et al., 2020).

3) A clarification question for the model setup: can the output weights be both excitatory and inhibitory?

Yes, output weights can be both excitatory and inhibitory. We clarified this in the methods.

4) In Figure 2a and b, the authors computed the energy for each point in the low-dimensional space. However, it seems to me this quantity is ill-defined, because a point in the low-dimensional space could correspond to a continuum of points in the full space, which may have different energies. It would be good to clarify how the energy in the low-d space was computed.

This observation is valid. For qualitative visualization purposes only, we created a grid of possible (x,y) coordinates in two-dimensional principal component space and performed an inverse PCA transform with *sklearn* function *inverse_transform*, which returns the unique orthogonal projection of the PC point onto native space. We clarified this in the Methods.

5) Figure 2j-m, the authors mentioned "The task cue was fixed to anti-DMS". Did the authors find the same results for pro-DMS task?

We thank the reivewer for this comment. We found similar findings for pro-DMS task (Response Fig. 11). We now include the figure as a supplementary figure (Supplementary Fig. 3).

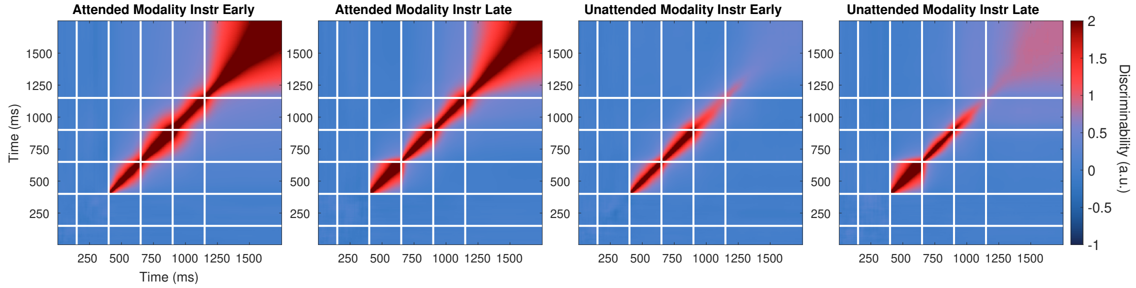

Response Fig. 11: **Temporal discriminability for the first stimulus identity when the task signal was fixed to pro-DMS**. For the attended modality (first two panels), the identity of the first stimulus was more robustly encoded throughout the trial duration than in the unattended modality (last two panels).

6) The authors mentioned that "However, the bifurcations driven by the identity of the first stimulus were not as well separated in the unattended modality (Fig. 2g)." Could the authors quantify the separation of the trajectories for attended vs unattended modalities?

We thank the reviewer for this helpful suggestion. To quantitatively assess the separation of neural trajectories for the attended versus unattended modalities shown in Fig. 2f and 2g, we now explicitly measured the Euclidean distance between latent trajectories corresponding to different first-stimulus identities.

Specifically, we randomly selected 10 latent trajectories within each of the four conditions defined by task and stimulus identity (pro, stim 1 = +1; pro, stim 1 = -1; anti, stim 1 = +1; anti, stim 1 = -1). We then computed the Euclidean distance in CEBRA latent space between trajectories corresponding to opposite stimulus identities separately for pro trials (pro, +1 vs. pro, -1) and anti trials (anti, +1 vs. anti, -1), restricted to the stimulus 1 time window.

This random subsampling procedure was repated 100 times to obtain a robust estimate of trajectory separation. The entire analysis was performed independently for the attended and unattended modalities (corresponding to Fig. 2f and 2g, respectively). Across networks and resampling iterations, the average latent-space distance between stimulus identities was significantly larger for the attended modality than for the unattended modality (Response Fig. 12), quantitatively confirming that stimulus-driven bifurcations are more strongly separated when the modality is attended.

We now included the following at lines 228 – 232:

"To quantify this effect, we measured the Euclidean distance between latent trajectories corresponding to opposite first-stimulus identities during the stimulus-1 period using repeated random subsampling. The average trajectory separation was significantly greater for the attended modality than for the unattended modality, confirming that stimulus-driven bifurcations are selectively

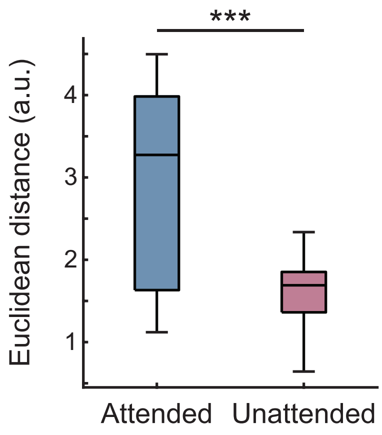

Response Fig. 12: **Quantification of latent trajectory separation for attended versus unattended modalities**. Boxplots show the Euclidean distance between CEBRA latent trajectories corresponding to opposite first-stimulus identities, computed during the stimulus 1 epoch. Distances were calculated separately for attended and unattended modalities by randomly subsampling latent trajectories across task conditions and repeating the procedure 100 times. Boxplot: central lines, median; bottom and top edges, lower and upper quartiles; whiskers, 1.5 × interquartile range; outliers are not plotted. $^{***}P < 0.001$ by two-sided Wilcoxon rank-sum test.

amplified by attention (Supplementary Fig. 2)."

7) A clarification question: in Figure 3h-k, when computing the centrality for E and I populations using Equation (8), did the authors just look at the correlations *within* E and I populations, or the correlation with the rest of the network (both E and I units)?

We computed centrality over all neurons, with the whole network, and then plotted the results separately for excitatory versus inhibitory neurons.

8) The authors mentioned that after lesioning I to I connections "the network exhibited a tendency to perform the pro-DMS task irrespective of the task cue presented." Is that a consistent bias observed across all the trained networks? If so, is there an explanation for why? I would imagine some networks have the opposite bias.

We thank the reviewer for raising this important point. We would like to clarify that the apparent bias toward the pro-DMS response is not a uniform or intrinsic bias across all trained networks. Rather, the observed effect depends on which subset of inhibitory-to-inhibitory ($I \to I$) connections is lesioned. Specifically, when inhibitory inputs to inhibitory units selective for the anti-DMS task cue are lesioned, anti-DMS signaling is selectively disrupted, leading to poorer performance on anti-DMS trials and an apparent tendency to perform the pro-DMS mapping. Conversely, when inhibitory connections to pro-DMS-selective inhibitory units are lesioned, networks show the opposite pattern, with selectively impaired performance on pro-DMS trials.

Thus, the behavioral bias reflects selective degradation of the lesioned task representation, rather than a global preference for one rule. This symmetry across task conditions is consistent across the trained networks and can be seen most clearly in Response Fig. 1 (bottom row), where lesioning selectively tuned inhibitory populations produces task-specific performance deficits in the corresponding condition.

9) In the two-module network, it looks like the sensory module sends exclusively excitatory connections to the non-sensory module, but the non-sensory module sends both excitatory and inhibitory

connections to the sensory module. Could the authors provide some justification for this asymmetry? I thought long-range connections are predominantly excitatory regardless of feedforward/feedback.

We thank the reviewer for raising this important point. We agree that long-range corticocortical projections are predominantly excitatory, particularly for feedforward signaling, and our model reflects this constraint by restricting sensory-to-non-sensory connections to be exclusively excitatory. However, accumulating experimental evidence indicates that top-down modulation can also be mediated by sparse but functionally significant long-range inhibitory influences (e.g., Melzer & Monyer, 2020; Schroeder et al., 2023). Motivated by these findings, we allowed the non-sensory module to exert both excitatory and inhibitory influences on the sensory module. We have revised the text to clarify this rationale and to emphasize that the modeled asymmetry reflects known differences between feedforward sensory transmission and feedback-mediated contextual control (lines 343–346):

"Given previous findings from animal studies showing that long-range feedforward projections are overwhelmingly excitatory, whereas top-down modulation can additionally engage sparse long-range inhibitory influences [31, 72], we trained our two-module RNNs to perform the one-modality DMS task without intermodule feedforward inhibitory projections."

10) In Figure 5c, the authors showed a line of fixed points in the state space for the two-module network. Could the authors clarify why a line attractor was found here but not in the one-module network? And what is the role of this line attractor in task performance (there does not seem to be anything that needs integration).

One possible interpretation for the putative line attractor in the 2-module condition is that neurons in each module represent bottom-up and top-down information asymmetrically in different periods of the task, leading to the existence of several invariant slow points stemming from different initial conditions. Specifically, in the modular architecture, sensory inputs act only on the sensory module, while instruction inputs and outputs are processed exclusively in the decision module. During the instruction period, many distinct decision-module states are compatible with identical sensory representations and identical future task performance, producing an invariant low-dimensional putative line attractor rather than a single isolated cluster of fixed points. Consistent with this interpretation, at the end of the instruction period, variance across slow points is significantly higher in decision-module neurons than in sensory neurons for both pro- and anti-task instructions (Response Fig. 13, KS test, $P < 0.001$), indicating that traversal along the putative line attractor is dominated by decision-module variability while sensory representations remain more tightly constrained. In contrast, the one-module network lacks this separation and converges to discrete fixed-point clusters. The line of slow points therefore could correspond to a family of functionally equivalent preparatory states that are weakly constrained during instruction and collapse once stimulus-driven dynamics begin.

11) Fig 6a, b: the authors only showed the lesion result for the sensory module but in the text they claimed the same result holds if they lesioned the connections in the non-sensory module. Could the authors show the results for the non-sensory module?

We thank the reviewer for pointing this out and apologize for the confusion caused by an error in the original figure labels.

In the previous version of the manuscript, the titles of Fig. 6a and Fig. 6b were incorrect. Specifically, Fig. 6a shows CEBRA neural trajectories for the intact two-module model, and Fig. 6b shows trajectories after lesioning inhibitory-to-inhibitory ($I \to I$) connections throughout the entire net-

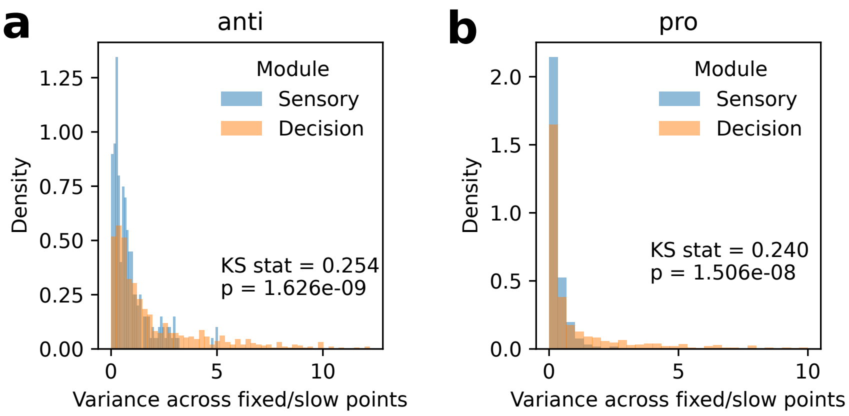

Response Fig. 13: **Slow point variance analysis**. **a.** Histogram displaying variance of neural activity in the anti-task condition, for each neuron, across all slow points, at the end of the instruction period for the 2-module RNN, containing sensory (blue) and decision (orange) modules. **b.** Same, for the pro-task condition.

work, including both the sensory and non-sensory modules, not only the sensory module as the original labels implied. We have now corrected these figure titles to accurately reflect the analyses.

In addition, in direct response to this comment (and the subsequent related comments), we have now explicitly included task-performance results for all possible lesion locations in the two-module architecture as supplementary figures (Supplementary Fig. 17–20): lesions within the sensory module, within the non-sensory module, from sensory to non-sensory modules, and from non-sensory to sensory modules. These results are now shown and discussed in the revised manuscript and accompanying supplementary figures, demonstrating that the key conclusions hold across lesion locations, with $I \rightarrow I$ disruptions in the non-sensory module producing particularly strong impairments.

12) Line 393-394: the authors mentioned "Lesioning other synaptic connections (i.e., E $\rightarrow$ E, E $\rightarrow$ I, I $\rightarrow$ E) did not lead to disruption of the top-down information encoding". Could the authors show these results?

Thank you for pointing this out. We now show all the results as supplementary figures (Supplementary Fig. 17–20). These figures are also shown below for your reference.

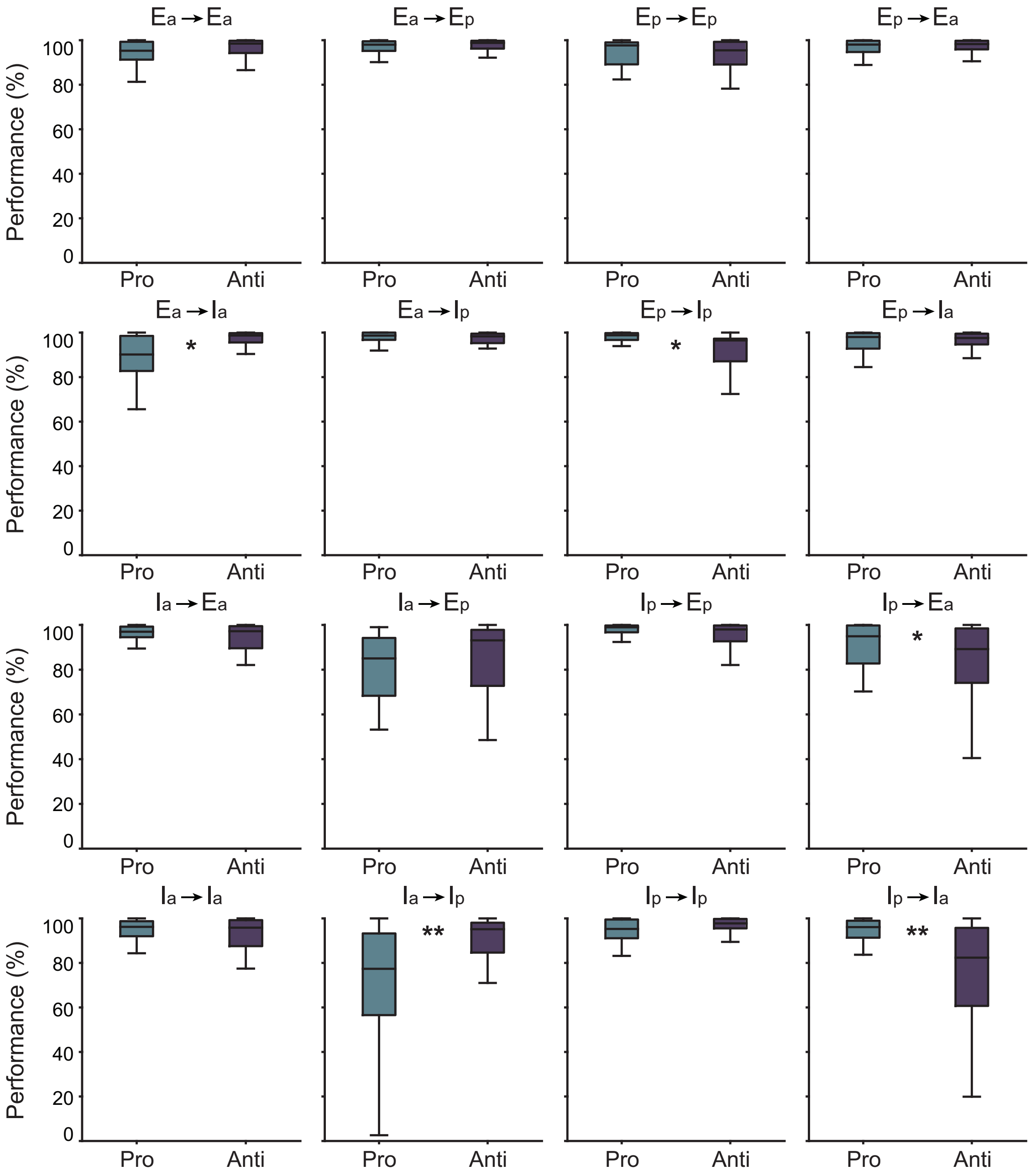

Response Fig. 14: **Perturbational analysis of excitatory and inhibitory synaptic connections within the sensory module in two-layer RNNs.** Task performance following selective lesioning of synaptic connections within the sensory module of two-layer RNNs trained to perform the one-modality task. Rows correspond to different connection classes ($E \to E$, $E \to I$, $I \to E$, $I \to I$), and columns show lesions applied to selectivity-matched or crossed populations (anti vs. pro). Lesions were implemented by reducing the corresponding synaptic weights by 50%, and performance is shown separately for anti and pro trials. Boxplot: central lines, median; bottom and top edges, lower and upper quartiles; whiskers, 1.5 $\times$ interquartile range; outliers are not plotted. $*P < 0.05$ and $**P < 0.01$ by two-sided Wilcoxon rank-sum test.

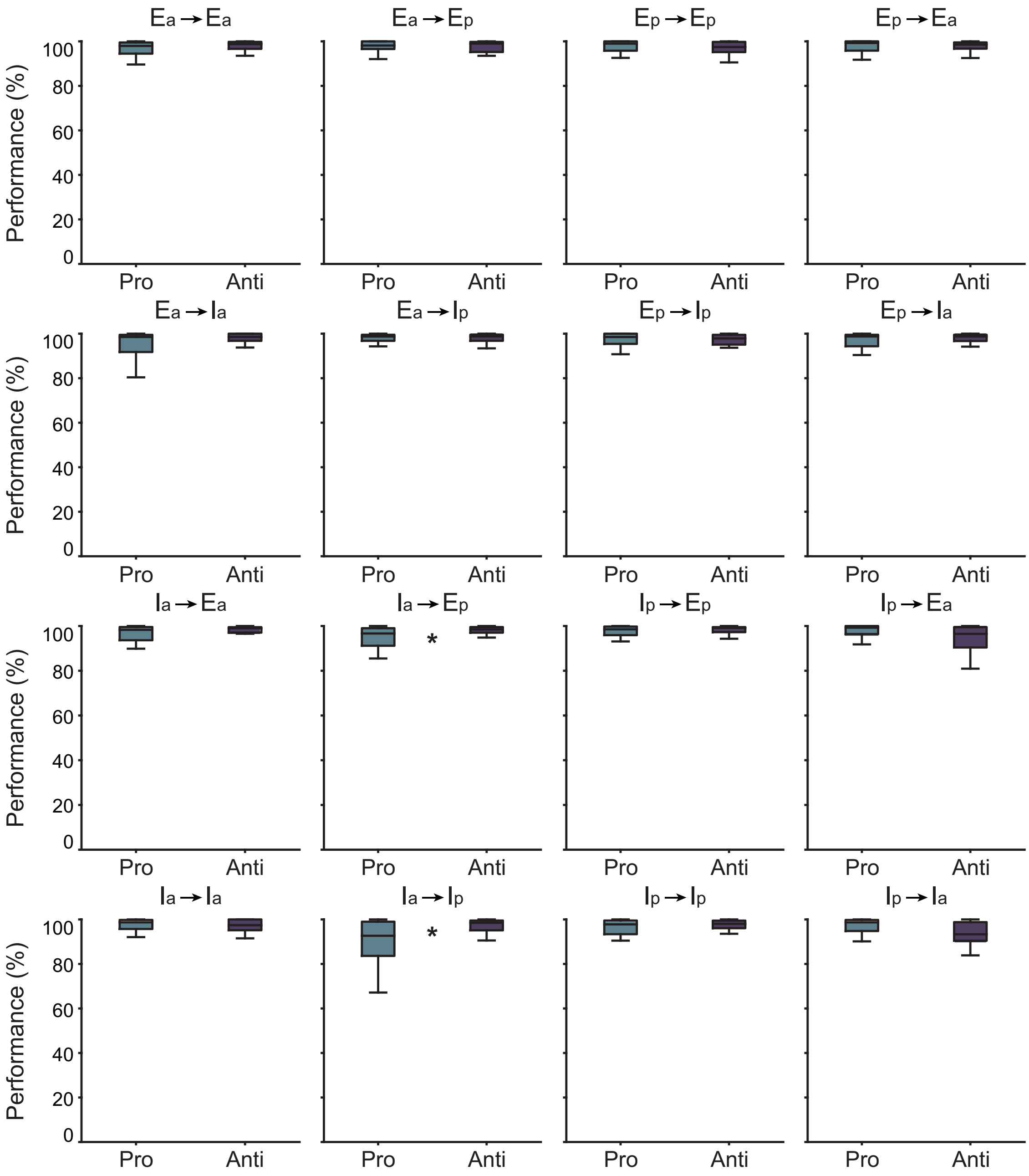

Response Fig. 15: **Perturbational analysis of excitatory and inhibitory synaptic connections from non-sensory module to sensory module in two-layer RNNs.** Task performance following selective lesioning of synaptic connections from the non-sensory module to the sensory module of two-layer RNNs trained to perform the one-modality task. Rows correspond to different connection classes ($E \to E$, $E \to I$, $I \to E$, $I \to I$), and columns show lesions applied to selectivity-matched or crossed populations (anti vs. pro). Lesions were implemented by reducing the corresponding synaptic weights by 50%, and performance is shown separately for anti and pro trials. Boxplot: central lines, median; bottom and top edges, lower and upper quartiles; whiskers, $1.5 \times$ interquartile range; outliers are not plotted. $^*P < 0.05$ by two-sided Wilcoxon rank-sum test.

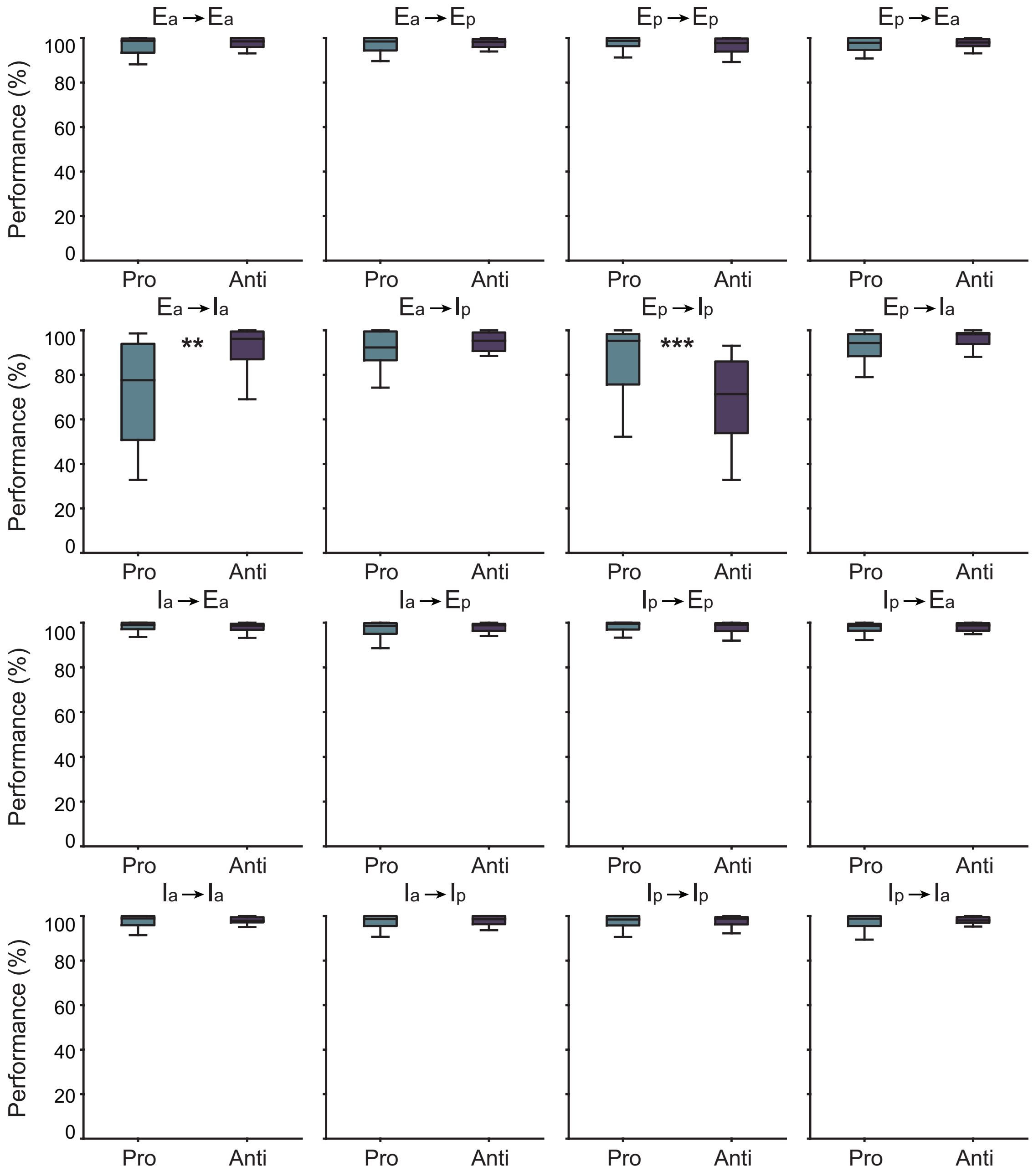

Response Fig. 16: **Perturbational analysis of excitatory and inhibitory synaptic connections from sensory module to non-sensory module in two-layer RNNs.** Task performance following selective lesioning of synaptic connections from the sensory module to the non-sensory module of two-layer RNNs trained to perform the one-modality task. Rows correspond to different connection classes ($E \rightarrow E$, $E \rightarrow I$, $I \rightarrow E$, $I \rightarrow I$), and columns show lesions applied to selectivity-matched or crossed populations (anti vs. pro). Lesions were implemented by reducing the corresponding synaptic weights by 50%, and performance is shown separately for anti and pro trials. Boxplot: central lines, median; bottom and top edges, lower and upper quartiles; whiskers, 1.5 × interquartile range; outliers are not plotted. $^{**}P < 0.01$ and $^{***}P < 0.001$ by two-sided Wilcoxon rank-sum test.

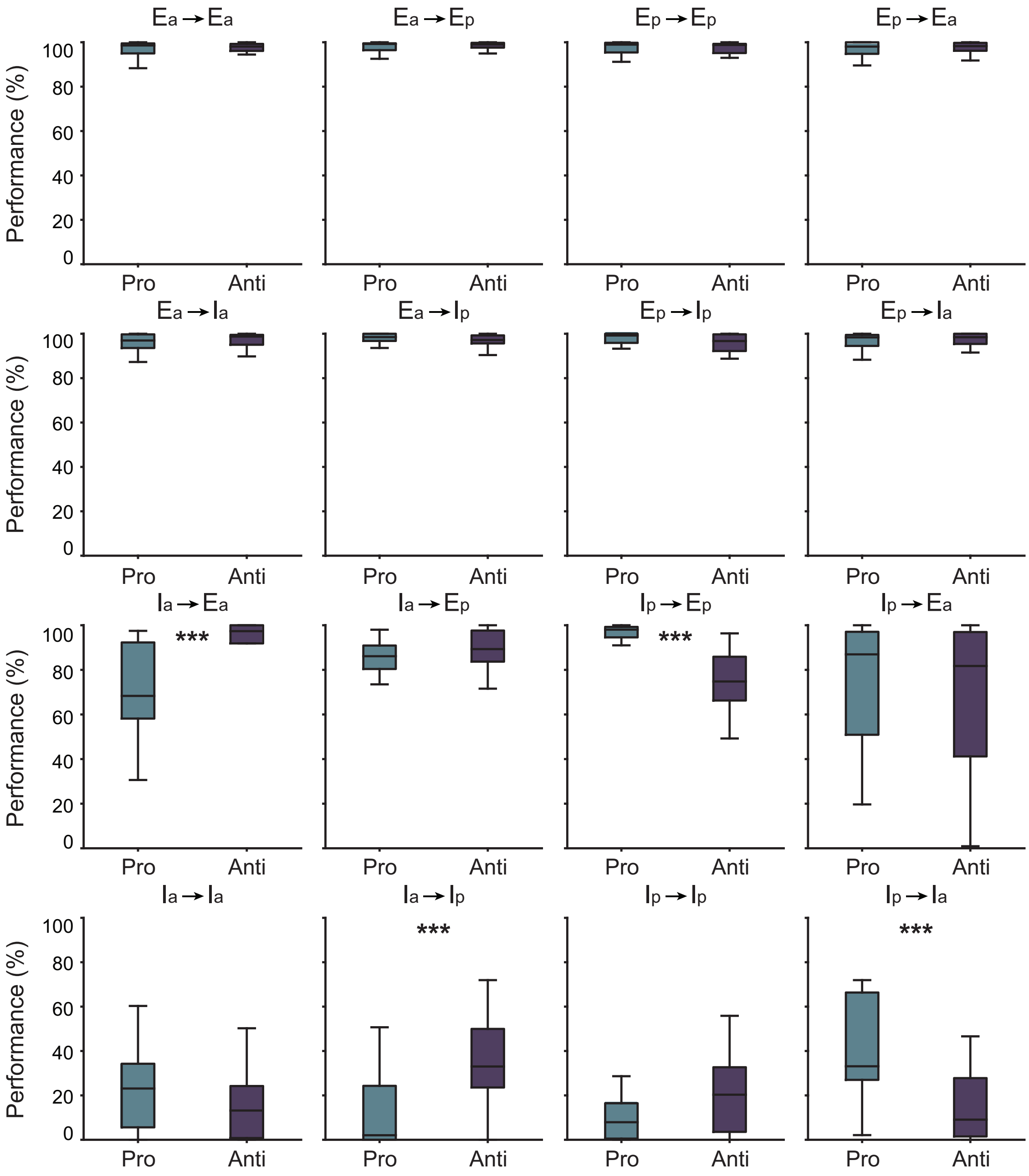

Response Fig. 17: **Perturbational analysis of excitatory and inhibitory synaptic connections within non-sensory module in two-layer RNNs.** Task performance following selective lesioning of synaptic connections within the non-sensory module of two-layer RNNs trained to perform the one-modality task. Rows correspond to different connection classes ($E \to E$, $E \to I$, $I \to E$, $I \to I$), and columns show lesions applied to selectivity-matched or crossed populations (anti vs. pro). Lesions were implemented by reducing the corresponding synaptic weights by 50%, and performance is shown separately for anti and pro trials. Boxplot: central lines, median; bottom and top edges, lower and upper quartiles; whiskers, 1.5 × interquartile range; outliers are not plotted. $^{***}P < 0.001$ by two-sided Wilcoxon rank-sum test.

13) The analyses in Figure 6c-f seem incomplete. It would be good if the authors could show the lesion results for all types of connections (I to I, E to E, I to E and E to I).

We thank the reviewer for this comment. Please see our response to the previous comment, where we present the corresponding results and describe the complete lesioning analyses across all synaptic connection types.

14) Line 408-409: the authors argued that "it is likely that I $\rightarrow$ E connections play a significant role in transmitting top-down information from the non-sensory module to the sensory module", but at the same time they said in line 402-403 that "lesioning feedback inhibitory connections had minimal influence on the task performance (Fig. 6e, f)." Aren't these two statements contradictory?

This observation is valid. For consistency, we amended our text by removing the claim that it is likely that I to E connections play a significant role in transmitting top-down information to the sensory module.

15) Line 405-407: the authors mentioned that "what is even more unexpected is that the top-down information is transmitted to the sensory module via feedback connections and encoded by I $\rightarrow$ I connections within the sensory module (Fig. 6a)". Because of the lack of control analyses, I think this statement is not well-supported based on the evidence in the current manuscript.

We thank the reviewer for this comment. In response to this concern, we have now included the full set of control lesion analyses in the two-module RNNs (Response Fig. 14–17) as supplementary figures, and we explicitly refer to these results in the revised text.

16) Lines 425-427: "this implies that any observed difference in figure versus ground coding within V1 neurons representing these receptive fields would stem from coding dependent on top-down feedback" - could it be due to lateral connections within V1 as well?

Previous evidence indicates these top-down signals do originate from areas that are downstream from V1 in the visual processsing hierarchy. We added two references to clarify this point. Lamme and Roelfsema, 2000 and Klink et al., 2017 lay the groundwork for how recurrent feedback from higher visual areas inform neural activity in V1, while Kirchberger et al., 2021 confirms causally that higher visual areas are the source of top-down signals into V1, considering the topographical organization of V1 receptive fields. We adapted the manuscript to discuss these results in a way that do not preclude the theoretical possibility of some lateral connections in V1.

17) Lines 439-442: the authors used the VIP to SST connections in the neural dataset as a proxy for the I to I connections in the model. However, there are many other I to I connections in the brain, like PV to PV, SST to PV, and SST to VIP. The authors need to clarify why VIP to SST connections correspond to the I to I connections in their model or at least mention this caveat.

The canonical VIP-SST disinhibitory circuit in cortex is a natural candidate for the biological implementation of the top-down regulatory mechanism elicited by inhibitory-inhibitory connections that arose in our artificial models because VIP neurons are known to receive long-range feedback from frontal areas. Therefore, VIP neurons are flexibly modulated by task demands, while inhibiting SST expressing neurons, which are inhibitory neurons themselves. However, it is valid to acknowledge the caveat that other I to I connections such as the ones cited exist in sensory circuits, and we altered the text to acknowledge this.

18) Figure 7f: for a better correspondence with the neural data in panel c, it would be good to (1) reverse the color, and (2) plot the same quantity on the y axis (either use decoding accuracy or discriminability).

We thank the reviewer for this helpful suggestion. To improve the correspondence between the model results in Fig. 7f and the neural data shown in Fig. 7c, we have made two changes as suggested by the reviewer. First, we reversed the color scheme in Fig. 7f to match that used in the neural data. Second, we replaced the temporal discriminability measure with linear SVM decoding accuracy, so that both panels now report the same quantity on the y-axis. We have now replaced our original Fig. 7f with the below figure and updated the corresponding figure caption.

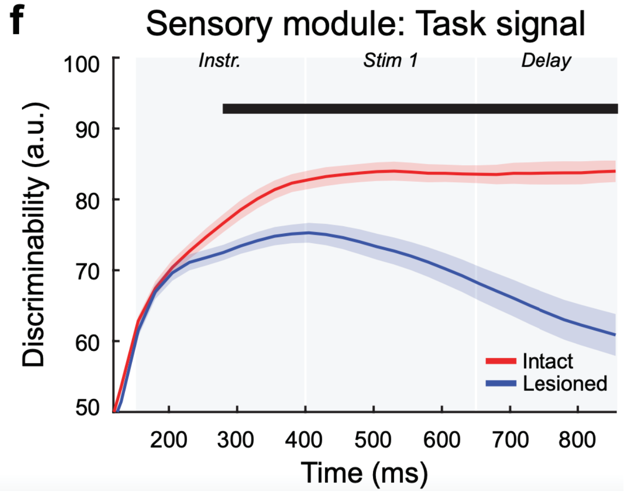

Response Fig. 18: **SVM decoding of task cue identity in the sensory module**. Mean linear SVM decoding accuracy of task cue identity (pro vs. anti) computed from sensory module population activity across all 20 trained two-module RNNs. Decoding was performed using sliding time windows with cross-validated train-test splits and was averaged first across neurons within the sensory module and then across models; shaded regions denote ± SEM. Red trace shows decoding accuracy for intact networks, while blue trace shows accuracy after removal of inhibitory-to-inhibitory ($I \rightarrow I$) connections within the sensory module. Thick black horizontal line denotes time points at which decoding accuracy differed significantly between intact and lesioned networks ($P < 0.05$, permutation test).

We have also revised the corresponding results section: (lines 507–513):

"Performing a time-resolved linear SVM decoding analysis on the sensory module across all 20 trained two-module RNNs revealed that the task cue identity could be reliably decoded from sensory population activity, providing additional confirmation of top-down modulation imposed by the non-sensory module (Fig. 7f; see *Methods*). When inhibitory-to-inhibitory ($I \rightarrow I$) connections within the sensory module were removed, task cue decoding accuracy was markedly reduced compared to the intact network (Fig. 7f), indicating that these disinhibitory connections play a critical role in enabling reliable top-down information encoding within the sensory circuitry."

We have also included additional details in the Methods section (lines 1109–1117):

"In parallel to the neural data analysis, we applied an analogous linear SVM decoding framework to activity from the sensory module of the trained two-module RNNs. For each model, task cue identity (pro vs. anti) was decoded from population activity using a time-resolved linear SVM. Neural activity was first averaged within sliding temporal windows (window size: 50 ms; step size: 25 ms) to generate feature vectors for each trial and time window. Classifiers were trained using a random 70% subset of trials and evaluated on the remaining 30% held-out trials, and this procedure was repeated across 100 random train-test splits. Decoding accuracy was computed independently

for each neuron and time window, then averaged across neurons within the sensory module and across trained models."

19) The effect in neural data (Figure 7c) is very small and very transient, compared to the model. Could the authors comment on that?

To address this point, we fully revisited the linear SVM decoding analysis of optogenetic silencing of VIP neurons within V1. To further align our analysis to Kirchberger et al. 2021, instead of selecting trials naively from the entire dataset, we restricted our analysis to the subset of orientation figure-ground effect trials. This subset was shown in the original manuscript (Kirchberger et al., 2021) to display the clearest figure-ground modulation in V1 neural populations.

Additionally, we performed a one-sided non-parametric test to determine whether the decoding performance was larger in the laser off condition, exclusively focused on the laser activation time window (from 0.25 s to 0.5 s following stim onset). Concretely, we are now analyzing a single 250 ms time window as a whole instead of testing several narrower time bins, which increased the robustness of the analysis. We argue this created a clearer picture in favor of the effect of optogenetic I-I silencing in disrupting top-down coding in V1, exclusively while the laser is turned on. The new results are displayed in the new Figure 7c, also included here. We have also amended the methods and results sections to reflect these changes.

20) Figure 8: I had a hard time seeing how the analyses on the retro-cue condition fit into the flow of the manuscript. Could the authors motivate a bit more the purpose of looking at retro-cue condition?

We agree with the reviewer that the retro-cue section would benefit from clearer motivation. The goal of introducing the retro-cue condition was to test whether the inhibitory-to-inhibitory mechanisms identified in the preceding sections generalize beyond fixed attentional demands, to scenarios in which stimulus relevance is revealed dynamically after sensory information has already been encoded. To make this point clear, we now revised the following sentence (lines 520–523):

"Identifying relevant sensory stimuli and determining the timing of their relevance is often dynamic in the real world. To better capture this, we modified the two-modality DMS task by introducing a retro-cue condition as illustrated in Figure 8a."

to:

"To test whether the disinhibitory circuit mechanisms identified above generalize to settings in which attentional relevance is specified after stimulus encoding, we introduced a retro-cue variant of the two-modality task as illustrated in Fig. 8a."

In addition, to more explicitly connect Fig. 8 to the core theme of the paper, namely the role of disinhibitory signaling in encoding and maintaining top-down information, we performed additional perturbational (lesioning) analyses analogous to those used throughout the manuscript.

To address this, we performed additional perturbational analyses in which we systematically lesioned each of the four synaptic connection types ($E \to E$, $E \to I$, $I \to E$, and $I \to I$) in RNNs trained on the modified two-modality task with the retro-cue condition. Across all three attention-cue timing conditions (early, late, and retro), lesioning inhibitory-to-inhibitory connections by a 50% reduction led to the most pronounced decrease in the temporal discriminability of the first stimulus, whereas lesioning the other three synaptic classes produced substantially weaker effects (Response Fig. 19). These results demonstrate that, despite the altered task demands imposed by the retro-cue condition, the network continues to rely on disinhibitory $I \to I$ circuitry to maintain stimulus representations over time.

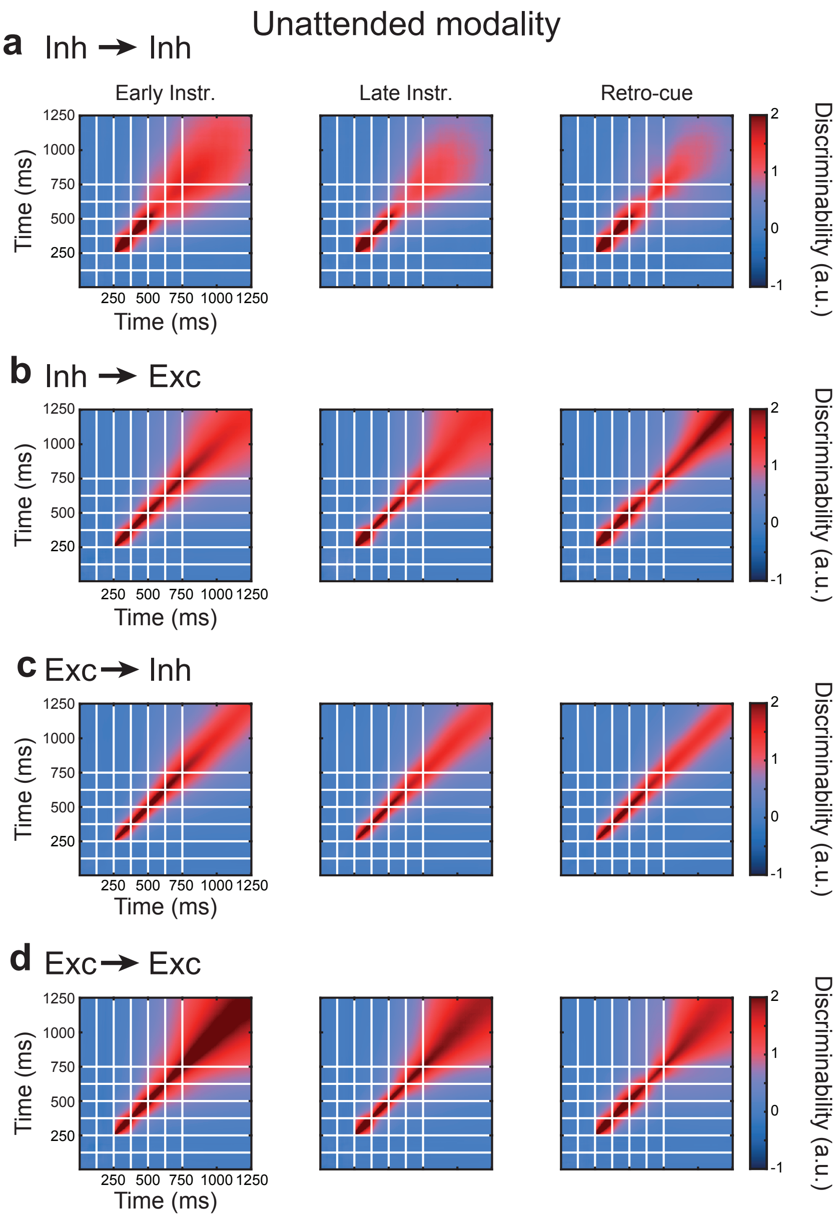

Response Fig. 19: **Effects of synaptic lesioning on unattended stimulus representations in two-module RNNs.** Average cross-temporal discriminability of the first stimulus identity of the unattended modality in two-module RNNs trained to perform the two-modality DMS task. Discriminability matrices are shown for trials in which the attention cue was presented early (left), late (middle), or as a retro-cue (right). Rows correspond to selective lesioning (50% reduction in synaptic weights) of different connection types: **a**. Inhibitory-to-inhibitory ($I \to I$). **b**. Inhibitory-to-excitatory ($I \to E$). **c**. Excitatory-to-inhibitory ($E \to I$). **d**. Excitatory-to-excitatory ($E \to E$) connections. White lines denote task epoch boundaries. Results are averaged across 20 trained RNNs.

Accordingly, we have updated Fig. 8 to explicitly include the $I \to I$ lesioning results, and present the full lesioning analyses (Response Fig. 19) in a new supplementary figure (Supplementary Fig. 21).

We have also revised the Results section to explicitly discuss these new analyses and clarify how the retro-cue findings in Fig. 8 relate to the broader disinhibitory mechanism identified throughout the study (lines 550–555):

"Carrying out systematic lesioning analyses revealed that reducing inhibitory-to-inhibitory ($I \to I$) connections led to a pronounced decrease in the temporal discriminability of the first stimulus (Fig. 8), whereas lesioning the other synaptic classes produced substantially weaker effects (Supplementary Fig. 21). These results demonstrate that, despite the altered task demands imposed by the retro-cue condition, stimulus representations continue to rely critically on disinhibitory $I \to I$ circuitry for their maintenance."

We also added the following sentence at lines 558–560:

"Notably, this shift in strategy preserves the same underlying circuit mechanism, with disinhibitory inhibitory-to-inhibitory interactions remaining essential for stabilizing stimulus representations under dynamically changing attentional demands."

21) Methods section, Equation (6): missing a square on the RHS.

We thank the reviewer for identifying this mistake, which now has been corrected.

22) Could the authors expand a bit more on the CEBRA method? For example, how is it different from doing PCA, if the authors were just using the unsupervised mode in CEBRA?

In contrast to PCA's fixed, linear dimensionality reduction that simply identifies orthogonal directions of maximum variance in the data, CEBRA in unsupervised (self-supervised) mode learns a nonlinear embedding using a contrastive training objective. PCA produces a linear projection based solely on covariance structure, without any learned model or explicit notion of similarity beyond variance maximization. By contrast, CEBRA (even in its unsupervised form) trains a neural network encoder with a contrastive loss (e.g., InfoNCE) that pulls similar pairs together and pushes dissimilar pairs apart, where similarity is defined via temporal proximity. This allows CEBRA to capture nonlinear structure and preserve relationships in the latent space that are not captured by PCA's variance-based orthogonal axes.

We added an abridged version of this explanation to the Results.
