# Supplementary material for: Disinhibitory signaling enables flexible coding of top-down information in cortical networks
Source: PLoS Biol. 2026 Jul 2;24(7):e3003831. doi: 10.1371/journal.pbio.3003831 (PMC13327264; doi:10.1371/journal.pbio.3003831)
Supplement: Attachment — Submitted filename: 25_Aquino_Kim_Response_Letter_R2.pdf [file pbio.3003831.s026.pdf]

Reviewer #1: The authors addressed all my questions carefully and extensively. Their paper is much improved and represents a useful contribution to the filed, of impressive depth and thoroughness.

We thank the reviewer for their thoughtful comments, which were critical for improving our manuscript.

Reviewer #2: The authors addressed my points, which were mostly concerning clarifications and discussion. It seems the authors put in a lot of effort to try addressing all reviewers' concerns.

We thank the reviewer for their thoughtful comments and are glad that our revisions have addressed their concerns. We appreciate the reviewer's recognition of our efforts to improve the clarity and discussion of the manuscript.

Reviewer #3: The authors addressed many of my concerns. I believe the manuscript is substantially strengthened. I have some remaining suggestions/questions below:

1. The authors discussed the functional benefits of disinhibition in the discussion section. I feel the discussion is good but still does not get to the core of my question. Of course, in a contextual task there needs to be some top-down input that amplify/stabilize the excitatory population that carries rule information, but why this job is better suited by a disinhibition motif than, say, just excitation? I know there is experimental data that support top-down modulation through disinhibition, but I would really appreciate it if the authors could think a bit more about why it is more advantageous than simply excitation, from a computational/theoretical perspective.

To address this, we leverage previous work in systems neuroscience which has specified the functional differences between E-E and I-I connections and the role of I-I connections in top-down control. Above and beyond being utilized for top-down regulation alone, inhibition plays a role in stabilizing cortical activity as a whole, as evidenced by the onset of seizures caused by loss of cortical inhibition [1]. Concretely, Sanzeni et al. [2] claim: "excitatory recurrent coupling alone can lead to unstable dynamics unless stabilized by inhibition. When recurrent connectivity is weak, excitatory cells can show stable firing rates independent of the activity of inhibitory cells. But in networks with strong recurrent connections, excitatory-to-excitatory (E-E) connections amplify responses so that the excitatory network is unstable if the firing rates of inhibitory neurons are kept fixed.". Indeed, models of excitatory networks with strong recurrent connections often require inhibitory-based stabilization to control self-amplification [3]. From the specific perspective of top-down control, disinhibitory feedback can act functionally as regulatory gating which modulates bottom-up circuits, as opposed to just re-writing sensory information coded by sensory pyramidal neurons [4, 5]. This allows top-down cortical feedback to select which parts of the sensory input matter more in a context-based manner. This type of long-range regulatory gating plays an especially important role in top-down attention-based gating of sensory inputs [6]. We have expanded on this specific topic accordingly in the Discussion.

2. The lesion results show the I→I local connections are particularly important for task performance. I think it is still worth trying to explain this phenomenon on a mechanistic level. For example, it would be helpful to look at the connectivity structure between the rule-selective E and I neurons. Are the I→I connections structured (I units more strongly inhibit other I units that prefer the opposite rule)? Are the other connections less structured? As shown the Roach et al paper that the authors cited, there are multiple connectivity motifs that can realize the same circuit function (in this case, selecting one population and suppressing the other). It would really get to the mechanistic level if the authors can show that the circuit connectivity matches the lesion results.

To address this, we analyzed the synaptic connectivity between rule-selective excitatory and in-

hibitory populations by comparing within-selectivity ("same"; anti → anti and pro → pro) and across-selectivity ("cross"; anti → pro and pro → anti) connection strengths across all trained RNNs (Response Fig. 1). Contrary to a purely cross-inhibitory competition motif, we found that I → I connections are strongly structured such that same-selectivity inhibition is significantly stronger (i.e., larger magnitude) than cross-selectivity inhibition ($p = 1.2\times10^{-4}$, paired Wilcoxon signed-rank test). Thus, inhibitory neurons preferentially inhibit other inhibitory neurons with the same rule preference.

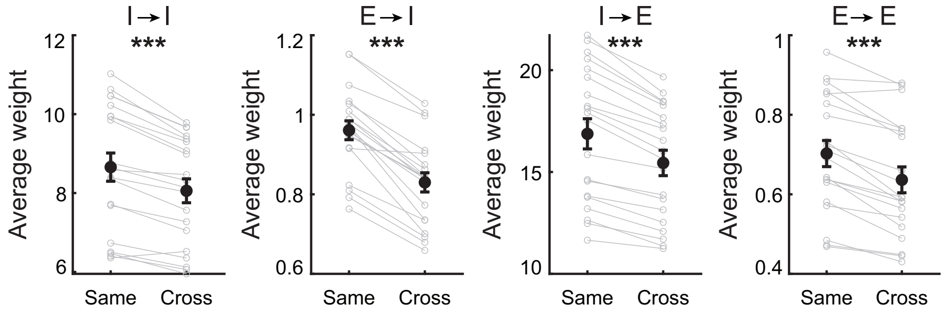

Response Fig. 1: **Structured connectivity across rule-selective populations**. Average synaptic weights (magnitude) between rule-selective populations across all trained one-module, one-modality RNNs ($n = 20$), separated into within-selectivity ("Same"; anti → anti and pro → pro) and across-selectivity ("Cross"; anti → pro and pro → anti) connections. Panels show results for inhibitory-to-inhibitory (I → I), excitatory-to-inhibitory (E → I), inhibitory-to-excitatory (I → E), and excitatory-to-excitatory (E → E) connections. Each gray line represents an individual network, and black markers denote mean ± s.e.m. ***$P < 0.001$ by paired Wilcoxon signed-rank test.

Importantly, we observed that this selectivity structure is present across connection types (I → I, I → E, E → I, and E → E), but is most functionally consequential in the I → I pathway. This suggests that while multiple connection motifs can, in principle, support selective routing (as noted by Roach et al. 2023), the network preferentially relies on structured inhibitory–inhibitory interactions.

Mechanistically, this pattern is consistent with a circuit in which I → I connections provide selective self-inhibition within rule-specific inhibitory subpopulations, thereby regulating the gain and stability of inhibitory activity within each rule channel [7, 8]. In this configuration, disinhibitory effects arise indirectly: by suppressing same-selectivity inhibitory neurons, the circuit can dynamically modulate inhibition onto the corresponding excitatory population, enabling flexible gating of task-relevant representations. Disrupting I → I connections therefore removes this intra-population inhibitory control, leading to dysregulated inhibitory activity and impaired task performance, consistent with the lesion results.

Taken together, these findings demonstrate that the functional importance of I→I connections arises from a structured, rule-dependent inhibitory architecture, providing a mechanistic link between connectivity organization and behavioral impairment following lesioning.

We now include Response Fig. 1 as Supplementary Fig. 6 and mention these results in lines 284–291:

"To understand the network structure underlying this increase in inhibitory centrality, we examined the connectivity patterns between rule-selective populations in the one-modality RNNs. Inhibitory neurons preferentially inhibited other inhibitory neurons with the same rule selectivity, with significantly stronger within-selectivity compared to across-selectivity I → I connections (Supplementary

Fig. 6). This structured motif was also observed across connection types, suggesting the emergence of rule-specific inhibitory subnetworks. Taken together, these results suggest a central role for inhibitory units in encoding task-specific top-down information, as well as stimulus identity."

3. Also, from Response Figure 15, lesioning the top-down long-range connections from the non-sensory to sensory module does not seem to affect performance much. Does this mean the top-down connections are not important for this task? This seems counter-intuitive.

We thank the reviewer for this important question. While Response Fig. 15 shows that partial perturbations (50% reduction) of long-range top-down connections from the non-sensory to the sensory module lead to relatively modest changes in performance, this does not imply that these connections are unimportant. To directly address this point, we performed an additional analysis in which top-down feedback connections were completely removed. Under this condition, removal of inhibitory top-down projections (I→I and I→E) resulted in a profound impairment in task performance (Response Fig. 2). These results demonstrate that top-down connections are necessary, but their contribution is not fully revealed by moderate perturbations, likely due to the robustness and redundancy of the network.

We interpret this as evidence that top-down feedback primarily acts to modulate and stabilize context-dependent representations in the sensory module, rather than directly driving behavior. As a result, partial reductions in these connections can be partially compensated by other pathways, whereas complete removal disrupts the network's ability to properly integrate contextual information.

4. The authors used the "inverse_transform" function from sklearn to compute the velocity and generate the potential. But mathematically, the inverse transform of PCA is not unique. So, this function must output a special inverse transform. Can the authors explain how the inverse_transform function work?

That is correct: mathematically, the inverse transform of PCA is not unique. However, among all the possible $X_{original}$ whose transform would be X, sklearn performs the following inverse transform, for a PCA rotation matrix W and dataset mean μ:

$$X_{original} = X_{pca} W^T + \mu \tag{1}$$

Any variance that was perpendicular to the principal components is treated as zero. Therefore, the reconstructed point is the unique orthogonal projection of the PC point onto native space, which is still lossy but the best approximation possible for visualization purposes. We explained this in more detail in our methods.

5. On the line attractor in Figure 5c: I understand that many decision states could be compatible with the task demand. But why these states form a line, rather than scattered around in a less ordered way? What does the location on the line represent? Do they just happen to lie on a line because of the dimensionality reduction method used? Or is it just by chance (is it reproducible across RNNs)?

We have re-run the fixed point search analysis for other RNN initialization seeds and found qualitatively that the formation of line-like attractors is not a consistent replicable feature of the search space. The most common observation was for fixed points to cluster along unordered point clouds as opposed to a line-like structure. For this reason, we prefer to not make any strong claims in this direction and added a caveat for this finding to the Results section.

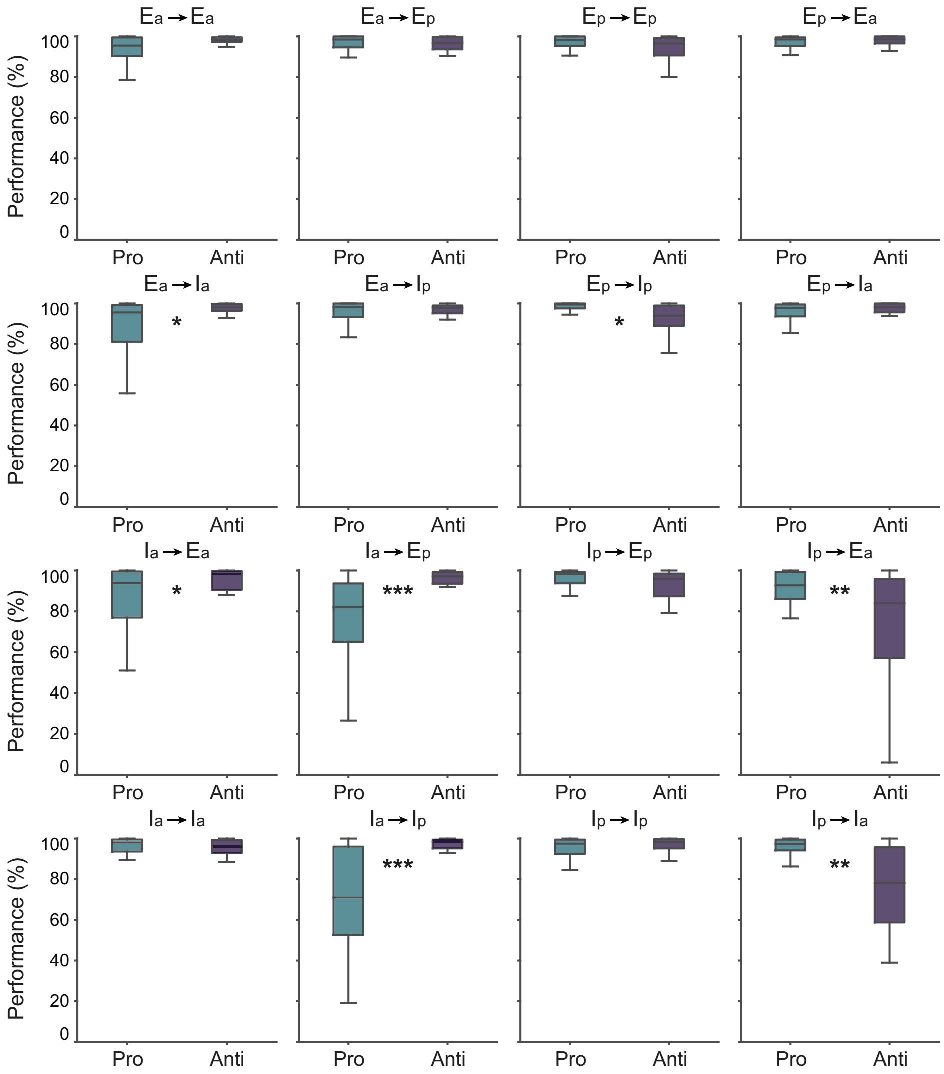

Response Fig. 2: **Perturbational analysis of excitatory and inhibitory synaptic connections from non-sensory module to sensory module in two-layer RNNs.** Task performance following selective removal of synaptic connections from the non-sensory module to the sensory module of two-layer RNNs trained to perform the one-modality task. Rows correspond to different connection classes ($E \rightarrow E$, $E \rightarrow I$, $I \rightarrow E$, $I \rightarrow I$), and columns show lesions applied to selectivity-matched or crossed populations (anti vs. pro). Lesions were implemented by completely removing the corresponding synaptic connections, and performance is shown separately for anti and pro trials. Boxplot: central lines, median; bottom and top edges, lower and upper quartiles; whiskers, 1.5 × interquartile range; outliers are not plotted. $*P < 0.05$ by two-sided Wilcoxon rank-sum test.

6. Title of Figure 6a and b seem to have not been corrected.

We thank the reviewer for pointing this out. This has been corrected now.

7. Response Figure 16, second row: it seems the excitatory long-range connections from the sensory to the non-sensory module are also helpful. Could the authors comment on that?

We thank the reviewer for this important observation. We agree that the results in Response Fig. 16 (second row) indicate that excitatory long-range projections from the sensory to the non-sensory module contribute to task performance. In particular, perturbations of E $\rightarrow$ I connections lead to moderate performance impairments, suggesting that excitatory drive from the sensory module helps recruit and shape activity in downstream inhibitory populations that are involved in context-dependent processing.

We revised lines 436–439 to mention these points:

"Lesioning other synaptic connections did not lead to disruption of top-down information encoding (Supplementary Fig. 17–20), although perturbations of $E \rightarrow I$ connections led to modest performance impairments, supporting role for long-range excitatory drive in recruiting inhibitory populations in higher-order areas."

8. I am a bit confused about the mice experiment. Do all the neurons in the recording have the same receptive field, and the image in the receptive was held constant throughout the session? Also, why does the bottom row of Figure 7a represent "control" trials? I thought these trials also contain the same image in the RF and different surroundings, so they can also be included in the decoding analysis.

We thank the reviewer for pointing this out and apologize for the confusion. The reviewer is correct that the neurons share the same receptive field, and the visual input within their receptive fields was held constant. To clarify this point, we have updated Fig. 7a to include receptive field schematics.

The bottom row of Fig. 7a represents control trials because, unlike the top row, these conditions do not require figure-ground segregation. In the control condition, the target is presented against a uniform gray background, making it directly detectable without the need to integrate contextual information from the surrounding visual field. In contrast, the top row requires figure-ground segregation, where the figure must be distinguished from a structured background based on contextual differences outside the receptive field.

9. Figure 7f: please change the label of y axis and the caption to "decoding accuracy" as opposed to "discriminability".

We thank the reviewer for identifying this issue. The y-axis label and figure caption have now been corrected to "decoding accuracy."

10. On the difference between the old and new Figure 7c: what is the difference between the trials that the authors sub-selected and the ones that are left out? It was not too clear from reading the authors' description.

In the original data manuscript, authors had tested 3 different figure-ground segregation schemes for visual gratings: orientation, phase, and texture. Behaviorally, authors found that orientation elicited the highest accuracy rates. Optogenetically, orientation was the one condition that elicited the highest differences in spike rates comparing laser on versus laser off conditions. Therefore, for our purposes of demonstrating the impact of optogenetic silencing on SVM decoding, we sub-selected only trials which had orientation as a top-down signaling cue and performed 5-fold cross-validation. Other than that, no further sub-selection was performed. We clarified this in the Methods.

11. Title of Figure 8 reads "Retro-cue signal forces the model to maintain both unattended and attended sensory signals". But from panels d-f, the model also remembers the unattended modality for early and late instruction trials. Is the discriminability maintained for longer for the retro-cue trials? Maybe the authors can plot the diagonal of the matrices versus time and compare between the three trial types.

We thank the reviewer for this helpful suggestion. To directly address this point, we computed and plotted the diagonal values of the cross-temporal discriminability matrices as a function of time for early, late, and retro-cue conditions (Response Fig. 3). While the unattended modality is indeed represented across all three conditions, the overall magnitude and temporal profile of discriminability were largely similar, with no substantial or sustained increase in the retro-cue condition. These results indicate that the key effect of the retro-cue manipulation is not simply prolonged maintenance of unattended information, but rather a shift in the network's encoding strategy, whereby both attended and unattended stimuli are more consistently retained across the trial.

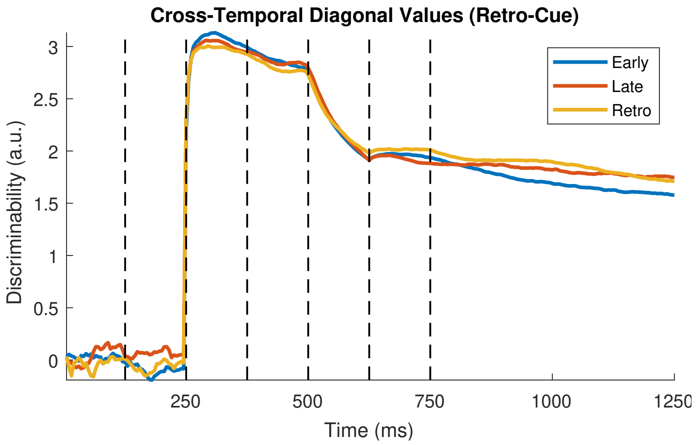

Response Fig. 3: **Temporal profile of discriminability across instruction conditions for the unattended modality**. Diagonal values of the cross-temporal discriminability matrices for the unattended modality are plotted as a function of time for early (blue), late (red), and retro-cue (yellow) conditions.

12. Related, in Response Figure 19, the difference between lesioning I→I and other connections seem to be mainly *after* stimulus presentation. At that time, the stimulus representation is being transformed, and therefore it is expected that the discriminability would go down. So, it seems insufficient to argue that I→I connections are particularly important for maintaining the stimulus. Again, I think it would be useful to look at how the zero-time-lag discriminability (the diagonal values) change over time and across conditions.

We thank the reviewer for this thoughtful comment. We agree that the largest differences between lesioning I → I and other connection types emerge after stimulus presentation, a period during which stimulus representations are actively transformed. Our primary goal in Response Fig. 19 was to assess how different synaptic pathways contribute to the evolution and transformation of stimulus representations over time, rather than isolating a purely maintenance-related signal. In this context, the pronounced reduction in discriminability following I → I lesioning specifically during post-stimulus periods indicates that these connections are important for preserving the structure of task-relevant representations as they are being dynamically updated.

Importantly, if the observed effects were solely due to generic transformation-related degradation, we would expect similar impairments across all connection types. Instead, the selective disruption observed for I $\rightarrow$ I lesioning suggests a specific functional role for inhibitory-inhibitory interactions in stabilizing and shaping representations during this transformation phase, rather than a nonspecific consequence of stimulus processing.

While a detailed analysis of diagonal elements could further refine the interpretation, we believe the current results already demonstrate that I $\rightarrow$ I connections play a disproportionate role in maintaining discriminability of task-relevant information during the period when representations are most vulnerable to degradation.

References

1. Steriade, M. & Contreras, D. Spike-wave complexes and fast components of cortically generated seizures. I. Role of neocortex and thalamus. *Journal of neurophysiology* **80,** 1439–1455 (1998).
2. Sanzeni, A., Akitake, B., Goldbach, H. C., Leedy, C. E., Brunel, N. & Histed, M. H. Inhibition stabilization is a widespread property of cortical networks. *Elife* **9,** e54875 (2020).
3. Ozeki, H., Finn, I. M., Schaffer, E. S., Miller, K. D. & Ferster, D. Inhibitory stabilization of the cortical network underlies visual surround suppression. *Neuron* **62,** 578–592 (2009).
4. Bertero, A., Garcia, C. & Apicella, A. J. Corticofugal VIP gabaergic projection neurons in the mouse auditory and motor cortex. *Frontiers in Neural Circuits* **15,** 714780 (2021).
5. Dellal, S., Zurita, H., Kruglikov, I., Valero, M., Abad-Perez, P., Geron, E., Meng, J. H., Prönneke, A., Hanson, J. L., Mir, E., *et al.* Inhibitory and disinhibitory VIP IN-mediated circuits in neocortex. *BioRxiv* (2025).
6. Zhang, S., Xu, M., Kamigaki, T., Hoang Do, J. P., Chang, W.-C., Jenvay, S., Miyamichi, K., Luo, L. & Dan, Y. Long-range and local circuits for top-down modulation of visual cortex processing. *Science* **345,** 660–665 (2014).
7. Pfeffer, C. K., Xue, M., He, M., Huang, Z. J. & Scanziani, M. Inhibition of inhibition in visual cortex: the logic of connections between molecularly distinct interneurons. *Nature neuroscience* **16,** 1068–1076 (2013).
8. Carandini, M. & Heeger, D. J. Normalization as a canonical neural computation. *Nature Reviews Neuroscience* **13,** 51–62 (Jan. 2012).
