# Supplementary material for: Disinhibitory signaling enables flexible coding of top-down information in cortical networks
Source: PLoS Biol. 2026 Jul 2;24(7):e3003831. doi: 10.1371/journal.pbio.3003831 (PMC13327264; doi:10.1371/journal.pbio.3003831)
Supplement: Peer review history

# Editor report: Decision Letter 0

**DOI:** 10.1371/journal.pbio.3003831.r001

**Authors:** Christian Schnell, PhD

11 Jul 2025

Dear Dr Rungratsameetaweemana,

Thank you for submitting your manuscript entitled "Disinhibitory signaling enables flexible coding of top-down information" for consideration as a Research Article by PLOS Biology.

Your manuscript has now been evaluated by the PLOS Biology editorial staff and I am writing to let you know that we would like to send your submission out for external peer review.

However, before we can send your manuscript to reviewers, we need you to complete your submission by providing the metadata that is required for full assessment. To this end, please login to Editorial Manager where you will find the paper in the 'Submissions Needing Revisions' folder on your homepage. Please click 'Revise Submission' from the Action Links and complete all additional questions in the submission questionnaire.

Once your full submission is complete, your paper will undergo a series of checks in preparation for peer review. After your manuscript has passed the checks it will be sent out for review. To provide the metadata for your submission, please Login to Editorial Manager (https://www.editorialmanager.com/pbiology) within two working days, i.e. by Jul 13 2025 11:59PM.

If your manuscript has been previously peer-reviewed at another journal, PLOS Biology is willing to work with those reviews in order to avoid re-starting the process. Submission of the previous reviews is entirely optional and our ability to use them effectively will depend on the willingness of the previous journal to confirm the content of the reports and share the reviewer identities. Please note that we reserve the right to invite additional reviewers if we consider that additional/independent reviewers are needed, although we aim to avoid this as far as possible. In our experience, working with previous reviews does save time.

If you would like us to consider previous reviewer reports, please edit your cover letter to let us know and include the name of the journal where the work was previously considered and the manuscript ID it was given. In addition, please upload a response to the reviews as a 'Prior Peer Review' file type, which should include the reports in full and a point-by-point reply detailing how you have or plan to address the reviewers' concerns.

During the process of completing your manuscript submission, you will be invited to opt-in to posting your pre-review manuscript as a bioRxiv preprint. Visit http://journals.plos.org/plosbiology/s/preprints for full details. If you consent to posting your current manuscript as a preprint, please upload a single Preprint PDF.

Feel free to email us at plosbiology@plos.org if you have any queries relating to your submission.

Kind regards,

Christian

Christian Schnell, PhD

Senior Editor

PLOS Biology

cschnell@plos.org

# Peer review: Decision Letter 1

**DOI:** 10.1371/journal.pbio.3003831.r002

**Authors:** Christian Schnell, PhD

20 Aug 2025

Dear Dr Rungratsameetaweemana,

Thank you for your patience while your manuscript "Disinhibitory signaling enables flexible coding of top-down information" was peer-reviewed at PLOS Biology. It has now been evaluated by the PLOS Biology editors, an Academic Editor with relevant expertise, and by several independent reviewers.

In light of the reviews, which you will find at the end of this email, we would like to invite you to revise the work to thoroughly address the reviewers' reports.

As you will see below, the reviewers agree that the study is well executed and provides important insights. They make a few recommendations to provide additional analyses, for better integration into the existing literature, better links to existing experimental data, and for improving the clarity and presentation.

Given the extent of revision needed, we cannot make a decision about publication until we have seen the revised manuscript and your response to the reviewers' comments. Your revised manuscript is likely to be sent for further evaluation by all or a subset of the reviewers.

In addition to these revisions, you will need to complete some formatting changes, which you will receive in a follow up email. A member of our team will be in touch with a set of requests shortly.

We expect to receive your revised manuscript within 3 months. Please email us (plosbiology@plos.org) if you have any questions or concerns, or would like to request an extension.

At this stage, your manuscript remains formally under active consideration at our journal; please notify us by email if you do not intend to submit a revision so that we may withdraw it.

**IMPORTANT - SUBMITTING YOUR REVISION**

Your revisions should address the specific points made by each reviewer. Please submit the following files along with your revised manuscript:

1. A 'Response to Reviewers' file - this should detail your responses to the editorial requests, present a point-by-point response to all of the reviewers' comments, and indicate the changes made to the manuscript.

*NOTE: In your point-by-point response to the reviewers, please provide the full context of each review. Do not selectively quote paragraphs or sentences to reply to. The entire set of reviewer comments should be present in full and each specific point should be responded to individually, point by point.

You should also cite any additional relevant literature that has been published since the original submission and mention any additional citations in your response.

2. In addition to a clean copy of the manuscript, please also upload a 'track-changes' version of your manuscript that specifies the edits made. This should be uploaded as a "Revised Article with Changes Highlighted" file type.

*Re-submission Checklist*

When you are ready to resubmit your revised manuscript, please refer to this re-submission checklist: https://plos.io/Biology_Checklist

To submit a revised version of your manuscript, please go to https://www.editorialmanager.com/pbiology/ and log in as an Author. Click the link labelled 'Submissions Needing Revision' where you will find your submission record.

Please make sure to read the following important policies and guidelines while preparing your revision:

*Published Peer Review*

Please note while forming your response, if your article is accepted, you may have the opportunity to make the peer review history publicly available. The record will include editor decision letters (with reviews) and your responses to reviewer comments. If eligible, we will contact you to opt in or out. Please see here for more details:

https://blogs.plos.org/plos/2019/05/plos-journals-now-open-for-published-peer-review/

*PLOS Data Policy*

Please note that as a condition of publication PLOS' data policy (http://journals.plos.org/plosbiology/s/data-availability) requires that you make available all data used to draw the conclusions arrived at in your manuscript. If you have not already done so, you must include any data used in your manuscript either in appropriate repositories, within the body of the manuscript, or as supporting information (N.B. this includes any numerical values that were used to generate graphs, histograms etc.). For an example see here: http://www.plosbiology.org/article/info%3Adoi%2F10.1371%2Fjournal.pbio.1001908#s5

*Blot and Gel Data Policy*

We require the original, uncropped and minimally adjusted images supporting all blot and gel results reported in an article's figures or Supporting Information files. We will require these files before a manuscript can be accepted so please prepare them now, if you have not already uploaded them. Please carefully read our guidelines for how to prepare and upload this data: https://journals.plos.org/plosbiology/s/figures#loc-blot-and-gel-reporting-requirements

*Protocols deposition*

To enhance the reproducibility of your results, we recommend that if applicable you deposit your laboratory protocols in protocols.io, where a protocol can be assigned its own identifier (DOI) such that it can be cited independently in the future. Additionally, PLOS ONE offers an option for publishing peer-reviewed Lab Protocol articles, which describe protocols hosted on protocols.io. Read more information on sharing protocols at https://plos.org/protocols?utm_medium=editorial-email&utm_source=authorletters&utm_campaign=protocols

Thank you again for your submission to our journal. We hope that our editorial process has been constructive thus far, and we welcome your feedback at any time. Please don't hesitate to contact us if you have any questions or comments.

Sincerely,

Christian

Christian Schnell, PhD

Senior Editor

PLOS Biology

cschnell@plos.org

------------------------------------

REVIEWS:

Reviewer #1: This paper uses extensive training and simulations with RNNs to investigate the interplay between task context and sensory coding. The authors describe how a disinhibitory motif arises as a result of training and study how this motif influences task performance and sensory representations. For that purpose, they use a few different variants of the RNN, as well as computational lesions. In addition, they use a publicly available experimental dataset, with which they carry out analyses that, as they report, substantiate their computational findings. Overall, the paper highlights the disinhibitory motif as a biologically plausible mechanism that is important for sensory processing, retaining of relevant information, and adaptive context integration.

I find the paper interesting, significant, and useful for the community. It is well written, and results are generally well substantiated by data and analyses. Below are several suggestions, which the authors might find useful for improving the paper.

*Would it make sense to carry out perturbational analysis of E->E and E->I connections? The analysis of inhibitory connections in Figs. 4 and 6 is interesting and useful, but doing the same for excitatory connections should reveal more about their relative roles, or at the very least establish sort of a baseline against which perturbations of inhibitory connections can be considered.

*It would be useful to provide example plots of activity in the RNNs - perhaps heat maps, with time on the x-axis, neuron ID on the y-axis, and the level of activity of each neuron indicated by color. I am sure readers would like to see how those plots look like for the different tasks, conditions, and models presented. They could be added to each figure where a new task or model is introduced. Also, maybe in Fig. 1, it would be good to show an example of such activity plot early in the training and after training.

*Lines 222-224: "Through dissection of the RNNs performing the one-modality and two-modality DMS tasks, we found that the optimized synaptic decay time constants for the excitatory units were significantly larger compared to the time constants of the inhibitory units". Also Fig. 3a,b and 5d,e.

I would recommend that the authors comment on how these observations compare with real biological data. One issue is that excitatory synaptic decay time constants tend to be shorter than inhibitory ones, e.g., in the mouse visual cortex, roughly by a factor of two. And the other is that typical values for these time constants are 10-30 ms (though both smaller and greater values are observed for some connection types), as opposed to 60-100 ms emerging in this work. See, for example, Campagnola et al., Science, 2022. While one should not demand too much biological realism from an RNN, it would be reasonable to explain the discrepancies and discuss why one should not worry about them too much.

*Line 445-446: "we observed a decrease in decoding accuracy following optogenetic silencing of V1 VIP neurons (p < 0.05, one-sided Kolmogorov-Smirnov test, Bonferroni corrected; Fig. 7c)".

Yes, but the difference is only 0.6 vs. 0.7 or so, and, more importantly, it is significant only during a very short time interval. To me, this seems like something one has to approach with some skepticism. I would recommend the authors to discuss the situation carefully and avoid making any substantial conclusions from this result.

*Fig. 8 and associated text - this seems like an interesting result, but it is not clear how it connects with the rest of the paper, which is primarily about disinhibitory signaling in coding of top-down information. Perhaps there is an opportunity for the authors to investigate mechanisms of operation of their RNN in this case and compare that with what they did in the rest of the paper.

*I would suggest adding several references to recent work on the structure and function of cortical disinhibitory circuits, such as that with a large experimental dataset from the Allen Institute.

Garrett et al. Stimulus novelty uncovers coding diversity in survey of visual cortex. BioRxiv (2025), https://doi.org/10.1101/2023.02.14.528085.

Piet et al. Behavioral strategy shapes activation of the Vip-Sst disinhibitory circuit in visual cortex. Neuron (2024), https://doi.org/10.1016/j.neuron.2024.02.008

Ito et al. Coordinated changes in a cortical circuit sculpt effects of novelty on neural dynamics. Cell Reports (2024), https://doi.org/10.1016/j.celrep.2024.114763

Schulz et al. The generation of cortical novelty responses through inhibitory plasticity. eLife (2021), https://doi.org/10.7554/eLife.65309

Wu et al. Regulation of circuit organization and function through inhibitory synaptic plasticity. Trends in Neurosciences (2022), https://doi.org/10.1016/j.tins.2022.10.006

Reviewer #2 (Katrin Franke signed her report): I believe this is a well-executed study that addresses an interesting question in sensory neuroscience. Specifically, understanding how context and top-down signals influence feedforward sensory processing is essential for explaining behavior, but this question is experimentally very challenging to approach. It typically requires selective silencing or manipulation of specific neural populations under well-controlled conditions, which can be difficult to achieve, especially at the level of precise microcircuits. Here, the authors take an elegant approach by using biologically constrained recurrent neural networks (RNNs) combined with circuit-level analyses of the model. This modeling framework allows for systematic and targeted investigation of circuit motifs—such as inhibitory-to-inhibitory connections—that may be difficult to test exhaustively in vivo. The inclusion of neural recordings from mouse visual cortex further strengthens the study by grounding the model findings in empirical data and demonstrating that similar circuit patterns may exist in the biological brain. While the results build on prior work and are generally consistent with current hypotheses about disinhibitory circuits and top-down modulation, the combination of computational and experimental approaches makes this study a valuable and well-integrated contribution to the field.

Major Points

Accessibility for Non-experts

The manuscript is well written and clearly motivated, but portions of the text and figures may be challenging for readers less familiar with computational modeling.

For example, Figure 1 would benefit from a brief explanation in the main text about how the RNNs are trained and constrained (e.g., Dale's principle), and why this matters.

In Figure 2a,b, interpreting the energy landscape plots was difficult, whereas the trajectory plots were more intuitive. It would help to clarify why both principal component space and latent space are used, how they differ, and how much variance each captures.

Figure Complexity and Redundancy

Several figures (e.g., Figures 3 and 4) contain many similar panels and analyses. While I appreciate the authors' thoroughness, these sections were difficult to parse.

Consider retaining only the most essential plots in the main figures, summarizing findings at a higher level, and moving more detailed analyses to the supplement.

Clarification of Quantitative Measures

It wasn't clear to me what the decoding plots represent, is this mean performance across all model units? Would be great to plot a measure of variability, e.g. through 5 or 10 fold cross-validation. Is this how the decoders were evaluated?

The concept of functional centrality, which is used to describe inhibitory units as network hubs, could also use a more intuitive explanation—what does this measure imply in terms of network function?

Experimental Validation (Figure 7)

The use of mouse V1 data to test model predictions is valuable. However, the effect of VIP silencing in Figure 7c appears relatively modest, especially when contrasted with clearer differences in trajectory structure (Figures 7d-e).

Some commentary on this discrepancy would be helpful, along with an indication of how much variance is explained by the latent space representations.

Task-Specificity and Generalization

The retro-cue condition is a nice addition that shows flexibility in the model's behavior. However, it raises the question of how specific the findings are to this particular task design.

I would encourage the authors to discuss whether the emergence of disinhibitory motifs might generalize to other forms of top-down modulation (e.g., reward-driven, predictive, or more naturalistic settings).

Biological Interpretation

The link between the modeled inhibitory units and known interneuron types (e.g., VIP→SST motifs) could be expanded. This would help readers better connect the RNN findings to cortical microcircuitry.

A more detailed discussion of biological implementation would be appreciated—especially for readers coming from an experimental background.

Reviewer #3: In this manuscript, the authors studied recurrent neural networks (RNNs) trained to perform context-dependent delay match-to-sample tasks, where the correct response depends on the combination of sensory stimulus, task cue (pro or anti), and in some cases, the attentional cue (attend to modality 1 or 2). The RNNs have separate excitatory and inhibitory populations, and the authors also developed two-module RNNs where each module receives input about sensory stimulus and contextual cue, respectively.

The authors mainly focused on the function of the inhibitory populations in the RNNs, in terms of 1) how their activity differentially represent the task context when compared with that of the excitatory population, and 2) the impact on task performance when inhibitory synapses are "lesioned" (set to lower strength) in the network. The authors concluded that inhibitory neurons, as well as inhibitory-to-inhibitory connections, play a more important role in representing contextual signals, which is consistent with recent experimental findings that VIP inhibitory neurons, which target other inhibitory neurons, mediate contextual information.

While this message is interesting and reported by an increasing number of experiments, I feel the analyses performed are not systematic and thorough enough to support this conclusion. For example, the authors showed that lesioning inhibitory-to-inhibitory (I to I) connections leads to an impairment in contextual behavioral performance, but did not do enough control analyses to show that lesioning other connections (E to E, E to I, I to E) leads to *less* performance deficit.

In addition, I would hope that a modeling study can reveal something beyond agreement with experimental data. The manuscript did not really touch on the deeper mechanistic questions, such as "why inhibitory neurons (and I to I connections) are more important in contextual tasks", or "what are the functional benefits of it".

Below are more detailed comments:

1)The authors showed that the task and attention modality could be decoded better in the inhibitory neurons compared to excitatory neurons (Fig 3c-g; Fig 5h, i). This is interesting but could the authors explore a bit more what is driving this difference. Is it simply because I cells have a higher firing rate, and therefore a higher signal-to-noise ratio?

2)Some comments related to the model lesion results in Figures 4 and 6

a.The authors wanted to conclude that I to I connections are most important in task performance by showing that lesioning I to I connections lead to a significant decrease in performance. However, no comparison was made e effect of lesioning E to I and E to E connections, as well as I to E connections in some cases. Without these control analyses, it is difficult to see that I to I connections play an *especially* important role.

b.The authors performed the lesion by decreasing the connection between *selective* neurons. To ensure a fair comparison, it would be better to look at the fraction of selective E and I neurons, to exclude the possibility that lesioning I to I connections has a larger effect simply because a larger fraction of connections was lesioned.

c.In Figures 4 and 6, lesioning Ia -> Ip connections impair anti-DMS trials more, and vice versa. Could the authors explore further why this is the case? Is it because Ip neurons inhibit Ea neurons? On the other hand, the opposite trend is true when it comes to modality 1 and 2 (Fig 4f, g). For example, lesioning the I_m1 -> I_m2 connections has a larger impact on performance for modality 1 trials). Why is this the case? Since the authors already have classified different neuron types, maybe it is worthwhile to look at the structure in the wiring between different neuron populations.

d.The authors performed the lesion by reducing the connection strength by 50%. Could the authors motivate why they chose 50% in particular? Is there some experimental evidence for choosing this number, or are the results robust across different levels of reduction?

3)If the authors indeed found that excitatory connections are less important (which I think still needs more systematic evaluation, as mentioned in the previous point), what are the functional benefits of inhibitory neurons and I to I connections being more important in contextual tasks?

4)The authors mentioned that in the one module network, the synaptic decay time constants for the excitatory units were significantly larger compared to those of the inhibitory units. The same is true for the non-sensory module of the two-module network, but the opposite was found for the sensory module. These results seem interesting and may warrants further exploration/discussion. For example, are they related to the functional difference between the E and I neurons in the model? Are there some functional benefits for this difference in time constants?

5)In the two-module network, the authors found that that the inhibitory connections are stronger in the non-sensory module compared to the sensory module, which is consistent with experimental data showing an increase in the strength of inhibition for higher-order cortices. It is nice that the model fits the data, but it would be nicer if the authors could offer some insights on any potential functional benefits this different in inhibition strength brings.

Minor comments:

1)The authors wrote in the introduction that "… However, these studies [of RNN modeling] primarily examine scenarios in with task goals and contextual cues remain fixed over time." This is not accurate. There are many studies that train RNNs to perform contextual tasks. Examples include

*Mante, V., Sussillo, D., Shenoy, K. V., & Newsome, W. T. (2013). Context-dependent computation by recurrent dynamics in prefrontal cortex. nature, 503(7474), 78-84.

*Yang, G. R., Joglekar, M. R., Song, H. F., Newsome, W. T., & Wang, X. J. (2019). Task representations in neural networks trained to perform many cognitive tasks. Nature neuroscience, 22(2), 297-306.

*Liu, Y., & Wang, X. J. (2024). Flexible gating between subspaces in a neural network model of internally guided task switching. Nature communications, 15(1), 6497.

2)Also in the introduction, the authors mentioned: "This results in temporally rich and highly dynamic decision-making, a phenomenon that has proven challenging to investigate in artificial neural network models, including RNNs". Could the authors provide some citations that support this claim?

3)A clarification question for the model setup: can the output weights be both excitatory and inhibitory?

4)In Figure 2a and b, the authors computed the energy for each point in the low-dimensional space. However, it seems to me this quantity is ill-defined, because a point in the low-dimensional space could correspond to a continuum of points in the full space, which may have different energies. It would be good to clarify how the energy in the low-d space was computed.

5)Figure 2j-m, the authors mentioned "The task cue was fixed to anti-DMS". Did the authors find the same results for pro-DMS task?

6)The authors mentioned that "However, the bifurcations driven by the identity of the first stimulus were not as well separated in the unattended modality (Fig. 2g)." Could the authors quantify the separation of the trajectories for attended vs unattended modalities?

7) A clarification question: in Figure 3h-k, when computing the centrality for E and I populations using Equation (8), did the authors just look at the correlations *within* E and I populations, or the correlation with the rest of the network (both E and I units)?

8)The authors mentioned that after lesioning I to I connections "the network exhibited a tendency to perform the pro-DMS task irrespective of the task cue presented." Is that a consistent bias observed across all the trained networks? If so, is there an explanation for why? I would imagine some networks have the opposite bias.

9)In the two-module network, it looks like the sensory module sends exclusively excitatory connections to the non-sensory module, but the non-sensory module sends both excitatory and inhibitory connections to the sensory module. Could the authors provide some justification for this asymmetry? I thought long-range connections are predominantly excitatory regardless of feedforward/feedback.

10)In Figure 5c, the authors showed a line of fixed points in the state space for the two-module network. Could the authors clarify why a line attractor was found here but not in the one-module network? And what is the role of this line attractor in task performance (there does not seem to be anything that needs integration).

11)Fig 6a, b: the authors only showed the lesion result for the sensory module but in the text they claimed the same result holds if they lesioned the connections in the non-sensory module. Could the authors show the results for the non-sensory module?

12)Line 393-394: the authors mentioned "Lesioning other synaptic connections (i.e., E → E, E → I, I → E) did not lead to disruption of the top-down information encoding". Could the authors show these results?

13) The analyses in Figure 6c-f seem incomplete. It would be good if the authors could show the lesion results for all types of connections (I to I, E to E, I to E and E to I).

14)Line 408-409: the authors argued that "it is likely that I → E connections play a significant role in transmitting top-down information from the non-sensory module to the sensory module", but at the same time they said in line 402-403 that "lesioning feedback inhibitory connections had minimal influence on the task performance (Fig. 6e, f)." Aren't these two statements contradictory?

15)Line 405-407: the authors mentioned that "what is even more unexpected is that the top-down information is transmitted to the sensory module via feedback connections and encoded by I → I connections within the sensory module (Fig. 6a)". Because of the lack of control analyses, I think this statement is not well-supported based on the evidence in the current manuscript.

16)Lines 425-427: "this implies that any observed difference in figure versus ground coding within V1 neurons representing these receptive fields would stem from coding dependent on top-down feedback" - could it be due to lateral connections within V1 as well?

17) Lines 439-442: the authors used the VIP to SST connections in the neural dataset as a proxy for the I to I connections in the model. However, there are many other I to I connections in the brain, like PV to PV, SST to PV, and SST to VIP. The authors need to clarify why VIP to SST connections correspond to the I to I connections in their model or at least mention this caveat.

18)Figure 7f: for a better correspondence with the neural data in panel c, it would be good to (1) reverse the color, and (2) plot the same quantity on the y axis (either use decoding accuracy or discriminability).

19)The effect in neural data (Figure 7c) is very small and very transient, compared to the model. Could the authors comment on that?

20)Figure 8: I had a hard time seeing how the analyses on the retro-cue condition fit into the flow of the manuscript. Could the authors motivate a bit more the purpose of looking at retro-cue condition?

21)Methods section, Equation (6): missing a square on the RHS.

22)Could the authors expand a bit more on the CEBRA method? For example, how is it different from doing PCA, if the authors were just using the unsupervised mode in CEBRA?

# Author comment: Author response to Decision Letter 2

**DOI:** 10.1371/journal.pbio.3003831.r003

26 Jan 2026

Attachment Submitted filename: 25_Aquino_Kim_Response_Letter_R1.pdf

# Peer review: Decision Letter 2

**DOI:** 10.1371/journal.pbio.3003831.r004

**Authors:** Christian Schnell, PhD

4 Mar 2026

Dear Dr Rungratsameetaweemana,

Thank you for your patience while we considered your revised manuscript "Disinhibitory signaling enables flexible coding of top-down information" for consideration as a Research Article at PLOS Biology. Your revised study has now been evaluated by the PLOS Biology editors, the Academic Editor and the original reviewers.

In light of the reviews, which you will find at the end of this email, we are pleased to offer you the opportunity to address the remaining points from the reviewers in a revision that we anticipate should not take you very long. We will then assess your revised manuscript and your response to the reviewers' comments with our Academic Editor aiming to avoid further rounds of peer-review, although we might need to consult with the reviewers, depending on the nature of the revisions.

In addition to these revisions, you may need to complete some formatting changes, which you will receive in a follow up email. A member of our team will be in touch with a set of requests shortly. If you do not receive a separate email within a few days, please assume that checks have been completed, and no additional changes are required.

We expect to receive your revised manuscript within 1 month. Please email us (plosbiology@plos.org) if you have any questions or concerns, or would like to request an extension.

At this stage, your manuscript remains formally under active consideration at our journal; please notify us by email if you do not intend to submit a revision so that we withdraw the manuscript.

**IMPORTANT - SUBMITTING YOUR REVISION**

Your revisions should address the specific points made by each reviewer. Please submit the following files along with your revised manuscript:

1. A 'Response to Reviewers' file - this should detail your responses to the editorial requests, present a point-by-point response to all of the reviewers' comments, and indicate the changes made to the manuscript.

*NOTE: In your point-by-point response to the reviewers, please provide the full context of each review. Do not selectively quote paragraphs or sentences to reply to. The entire set of reviewer comments should be present in full and each specific point should be responded to individually.

You should also cite any additional relevant literature that has been published since the original submission and mention any additional citations in your response.

2. In addition to a clean copy of the manuscript, please also upload a 'track-changes' version of your manuscript that specifies the edits made. This should be uploaded as a "Revised Article with Changes Highlighted " file type.

*Resubmission Checklist*

When you are ready to resubmit your revised manuscript, please refer to this resubmission checklist: https://plos.io/Biology_Checklist

To submit a revised version of your manuscript, please go to https://www.editorialmanager.com/pbiology/ and log in as an Author. Click the link labelled 'Submissions Needing Revision' where you will find your submission record.

Please make sure to read the following important policies and guidelines while preparing your revision:

*Published Peer Review*

Please note while forming your response, if your article is accepted, you may have the opportunity to make the peer review history publicly available. The record will include editor decision letters (with reviews) and your responses to reviewer comments. If eligible, we will contact you to opt in or out. Please see here for more details:

https://blogs.plos.org/plos/2019/05/plos-journals-now-open-for-published-peer-review/

*PLOS Data Policy*

Please note that as a condition of publication PLOS' data policy (http://journals.plos.org/plosbiology/s/data-availability) requires that you make available all data used to draw the conclusions arrived at in your manuscript. If you have not already done so, you must include any data used in your manuscript either in appropriate repositories, within the body of the manuscript, or as supporting information (N.B. this includes any numerical values that were used to generate graphs, histograms etc.). For an example see here: http://www.plosbiology.org/article/info%3Adoi%2F10.1371%2Fjournal.pbio.1001908#s5

*Blot and Gel Data Policy*

We require the original, uncropped and minimally adjusted images supporting all blot and gel results reported in an article's figures or Supporting Information files. We will require these files before a manuscript can be accepted so please prepare them now, if you have not already uploaded them. Please carefully read our guidelines for how to prepare and upload this data: https://journals.plos.org/plosbiology/s/figures#loc-blot-and-gel-reporting-requirements

*Protocols deposition*

To enhance the reproducibility of your results, we recommend that if applicable you deposit your laboratory protocols in protocols.io, where a protocol can be assigned its own identifier (DOI) such that it can be cited independently in the future. Additionally, PLOS ONE offers an option for publishing peer-reviewed Lab Protocol articles, which describe protocols hosted on protocols.io. Read more information on sharing protocols at https://plos.org/protocols?utm_medium=editorial-email&utm_source=authorletters&utm_campaign=protocols

Thank you again for your submission to our journal. We hope that our editorial process has been constructive thus far, and we welcome your feedback at any time. Please don't hesitate to contact us if you have any questions or comments.

Sincerely,

Christian

Christian Schnell, PhD

Senior Editor

PLOS Biology

cschnell@plos.org

----------------------------------------------------------------

REVIEWS:

Reviewer #1: The authors addressed all my questions carefully and extensively. Their paper is much improved and represents a useful contribution to the filed, of impressive depth and thoroughness.

Reviewer #2: The authors addressed my points, which were mostly concerning clarifications and discussion. It seems the authors put in a lot of effort to try addressing all reviewers' concerns.

Reviewer #3: The authors addressed many of my concerns. I believe the manuscript is substantially strengthened. I have some remaining suggestions/questions below:

1.The authors discussed the functional benefits of disinhibition in the discussion section. I feel the discussion is good but still does not get to the core of my question. Of course, in a contextual task there needs to be some top-down input that amplify/stabilize the excitatory population that carries rule information, but why this job is better suited by a disinhibition motif than, say, just excitation? I know there is experimental data that support top-down modulation through disinhibition, but I would really appreciate it if the authors could think a bit more about why it is more advantageous than simply excitation, from a computational/theoretical perspective.

2.The lesion results show the I->I local connections are particularly important for task performance. I think it is still worth trying to explain this phenomenon on a mechanistic level. For example, it would be helpful to look at the connectivity structure between the rule-selective E and I neurons. Are the I->I connections structured (I units more strongly inhibit other I units that prefer the opposite rule)? Are the other connections less structured? As shown the Roach et al paper that the authors cited, there are multiple connectivity motifs that can realize the same circuit function (in this case, selecting one population and suppressing the other). It would really get to the mechanistic level if the authors can show that the circuit connectivity matches the lesion results.

3.Also, from Response Figure 15, lesioning the top-down long-range connections from the non-sensory to sensory module does not seem to affect performance much. Does this mean the top-down connections are not important for this task? This seems counter-intuitive.

4.The authors used the "inverse_transform" function from sklearn to compute the velocity and generate the potential. But mathematically, the inverse transform of PCA is not unique. So, this function must output a special inverse transform. Can the authors explain how the inverse_transform function work?

5.On the line attractor in Figure 5c: I understand that many decision states could be compatible with the task demand. But why these states form a line, rather than scattered around in a less ordered way? What does the location on the line represent? Do they just happen to lie on a line because of the dimensionality reduction method used? Or is it just by chance (is it reproducible across RNNs)?

6.Title of Figure 6a and b seem to have not been corrected.

7.Response Figure 16, second row: it seems the excitatory long-range connections from the sensory to the non-sensory module are also helpful. Could the authors comment on that?

8.I am a bit confused about the mice experiment. Do all then neurons in the recording have the same receptive field, and the image in the receptive was held constant throughout the session? Also, why does the bottom row of Figure 7a represent "control" trials? I thought these trials also contain the same image in the RF and different surroundings, so they can also be included in the decoding analysis.

9.Figure 7f: please change the label of y axis and the caption to "decoding accuracy" as opposed to "discriminability".

10.On the difference between the old and new Figure 7c: what is the difference between the trials that the authors sub-selected and the ones that are left out? It was not too clear from reading the authors' description.

11.Title of Figure 8 reads "Retro-cue signal forces the model to maintain both unattended and attended sensory signals". But from panels d-f, the model also remembers the unattended modality for early and late instruction trials. Is the discriminability maintained for longer for the retro-cue trials? Maybe the authors can plot the diagonal of the matrices versus time and compare between the three trial types.

12.Related, in Response Figure 19, the difference between lesioning I->I and other connections seem to be mainly *after* stimulus presentation. At that time, the stimulus representation is being transformed, and therefore it is expected that the discriminability would go down. So, it seems insufficient to argue that I->I connections are particularly important for maintaining the stimulus. Again, I think it would be useful to look at how the zero-time-lag discriminability (the diagonal values) change over time and across conditions.

# Author comment: Author response to Decision Letter 3

**DOI:** 10.1371/journal.pbio.3003831.r005

27 Apr 2026

Attachment Submitted filename: 25_Aquino_Kim_Response_Letter_R2.pdf

# Editor report: Decision Letter 3

**DOI:** 10.1371/journal.pbio.3003831.r006

**Authors:** Christian Schnell, PhD

30 Apr 2026

Dear Dr Rungratsameetaweemana,

Thank you for your patience while we considered your revised manuscript "Disinhibitory signaling enables flexible coding of top-down information" for publication as a Research Article at PLOS Biology. This revised version of your manuscript has been evaluated by the PLOS Biology editors and the Academic Editor.

Based on our Academic Editor's assessment of your revision, we are likely to accept this manuscript for publication, provided you satisfactorily address the following data and other policy-related requests:

* We would like to suggest a different title to improve its accessibility for our broad audience:

Disinhibitory signaling enables flexible coding of top-down information in mouse visual cortex

* Please add the links to the funding agencies in the Financial Disclosure statement in the manuscript details.

* DATA POLICY:

You may be aware of the PLOS Data Policy, which requires that all data be made available without restriction: http://journals.plos.org/plosbiology/s/data-availability. For more information, please also see this editorial: http://dx.doi.org/10.1371/journal.pbio.1001797

Note that we do not require all raw data. Rather, we ask that all individual quantitative observations that underlie the data summarized in the figures and results of your paper be made available in one of the following forms:

1) Supplementary files (e.g., excel). Please ensure that all data files are uploaded as 'Supporting Information' and are invariably referred to (in the manuscript, figure legends, and the Description field when uploading your files) using the following format verbatim: S1 Data, S2 Data, etc. Multiple panels of a single or even several figures can be included as multiple sheets in one excel file that is saved using exactly the following convention: S1_Data.xlsx (using an underscore).

2) Deposition in a publicly available repository. Please also provide the accession code or a reviewer link so that we may view your data before publication.

Regardless of the method selected, please ensure that you provide the individual numerical values that underlie the summary data displayed in the following figure panels as they are essential for readers to assess your analysis and to reproduce it: 1C, 3AB, 4CDE, 5DEFG, 6CDEF, S2, S6-S13, S15, S16, S18-S21

NOTE: the numerical data provided should include all replicates AND the way in which the plotted mean and errors were derived (it should not present only the mean/average values).

Please also ensure that figure legends in your manuscript include information on where the underlying data can be found, and ensure your supplemental data file/s has a legend.

Please ensure that your Data Statement in the submission system accurately describes where your data can be found.

* CODE POLICY

Per journal policy, if you have generated any custom code during the course of this investigation, please make it available without restrictions. Please ensure that the code is sufficiently well documented and reusable, and that your Data Statement in the Editorial Manager submission system accurately describes where your code can be found. More information on our Code Policy, what and how to share can be found here: https://journals.plos.org/plosbiology/s/code-availability

Please note that we cannot accept sole deposition of code in GitHub, as this could be changed after publication. However, you can archive this version of your publicly available GitHub code to Zenodo. Once you do this, it will generate a DOI number, which you will need to provide in the Data Accessibility Statement (you are welcome to also provide the GitHub access information). See the process for doing this here: https://docs.github.com/en/repositories/archiving-a-github-repository/referencing-and-citing-content

As you address these items, please take this last chance to review your reference list to ensure that it is complete and correct. If you have cited papers that have been retracted, please include the rationale for doing so in the manuscript text, or remove these references and replace them with relevant current references. Any changes to the reference list should be mentioned in the cover letter that accompanies your revised manuscript.

In addition to these revisions, you may need to complete some formatting changes, which you will receive in a follow up email. A member of our team will be in touch with a set of requests shortly. If you do not receive a separate email within a few days, please assume that checks have been completed, and no additional changes are required.

We expect to receive your revised manuscript within two weeks.

To submit your revision, please go to https://www.editorialmanager.com/pbiology/ and log in as an Author. Click the link labelled 'Submissions Needing Revision' to find your submission record. Your revised submission must include the following:

- a cover letter that should detail your responses to any editorial requests, if applicable, and whether changes have been made to the reference list

- a Response to Reviewers file that provides a detailed response to the reviewers' comments (if applicable, if not applicable please do not delete your existing 'Response to Reviewers' file.)

- a track-changes file indicating any changes that you have made to the manuscript.

NOTE: If Supporting Information files are included with your article, note that these are not copyedited and will be published as they are submitted. Please ensure that these files are legible and of high quality (at least 300 dpi) in an easily accessible file format. For this reason, please be aware that any references listed in an SI file will not be indexed. For more information, see our Supporting Information guidelines:

https://journals.plos.org/plosbiology/s/supporting-information

*Published Peer Review History*

Please note that you may have the opportunity to make the peer review history publicly available. The record will include editor decision letters (with reviews) and your responses to reviewer comments. If eligible, we will contact you to opt in or out. Please see here for more details:

https://plos.org/published-peer-review-history/

*Press*

Should you, your institution's press office or the journal office choose to press release your paper, please ensure you have opted out of Early Article Posting on the submission form. We ask that you notify us as soon as possible if you or your institution is planning to press release the article.

*Protocols deposition*

To enhance the reproducibility of your results, we recommend that if applicable you deposit your laboratory protocols in protocols.io, where a protocol can be assigned its own identifier (DOI) such that it can be cited independently in the future. Additionally, PLOS ONE offers an option for publishing peer-reviewed Lab Protocol articles, which describe protocols hosted on protocols.io. Read more information on sharing protocols at https://plos.org/protocols?utm_medium=editorial-email&utm_source=authorletters&utm_campaign=protocols

Please do not hesitate to contact me should you have any questions.

Sincerely,

Christian

Christian Schnell, PhD

Senior Editor

cschnell@plos.org

PLOS Biology

# Editor report: Decision Letter 4

**DOI:** 10.1371/journal.pbio.3003831.r007

**Authors:** Christian Schnell, PhD

18 May 2026

Dear Dr Rungratsameetaweemana,

Thank you for the submission of your revised Research Article "Disinhibitory signaling enables flexible coding of top-down information in cortical networks" for publication in PLOS Biology. On behalf of my colleagues and the Academic Editor, Adam Kohn, I am pleased to say that we can in principle accept your manuscript for publication, provided you address any remaining formatting and reporting issues. These will be detailed in an email you should receive within 2-3 business days from our colleagues in the journal operations team; no action is required from you until then. Please note that we will not be able to formally accept your manuscript and schedule it for publication until you have completed any requested changes.

While you attend to those requests, please also ensure that all corresponding figure legends in your manuscript include information on where the underlying data can be found, for example: "Source data can be found in Zenodo at 10.5281/zenodo.20148306."

Please take a minute to log into Editorial Manager at http://www.editorialmanager.com/pbiology/, click the "Update My Information" link at the top of the page, and update your user information to ensure an efficient production process.

PRESS

We frequently collaborate with press offices. If your institution or institutions have a press office, please notify them about your upcoming paper at this point, to enable them to help maximize its impact. If the press office is planning to promote your findings, we would be grateful if they could coordinate with biologypress@plos.org. If you have previously opted in to the early version process, we ask that you notify us immediately of any press plans so that we may opt out on your behalf.

We also ask that you take this opportunity to read our Embargo Policy regarding the discussion, promotion and media coverage of work that is yet to be published by PLOS. As your manuscript is not yet published, it is bound by the conditions of our Embargo Policy. Please be aware that this policy is in place both to ensure that any press coverage of your article is fully substantiated and to provide a direct link between such coverage and the published work. For full details of our Embargo Policy, please visit http://www.plos.org/about/media-inquiries/embargo-policy/.

Thank you again for choosing PLOS Biology for publication and supporting Open Access publishing. We look forward to publishing your study.

Sincerely,

Christian

Christian Schnell, PhD

Senior Editor

PLOS Biology

cschnell@plos.org
